# Surface Modifiers on Composite Particles for Direct Compaction

**DOI:** 10.3390/pharmaceutics14102217

**Published:** 2022-10-18

**Authors:** Fu-Cai Chen, Wen-Jun Liu, Wei-Feng Zhu, Ling-Yu Yang, Ji-Wen Zhang, Yi Feng, Liang-Shan Ming, Zhe Li

**Affiliations:** 1 Key Laboratory of Preparation of Modern TCM, Ministry of Education, Jiangxi University of Chinese Medicine, Nanchang 330004, China; 2 Jiangzhong Pharmaceutical Co., Ltd., Nanchang 330049, China; 3 Shanghai Institute of Materia Medica, Chinese Academy of Sciences, Shanghai 201203, China; 4 Engineering Research Center of Modern Preparation Technology of TCM of Ministry of Education, Shanghai University of Traditional Chinese Medicine, Shanghai 201203, China

**Keywords:** direct compaction, particle structure, surface modifier, co-processing

## Abstract

Direct compaction (DC) is considered to be the most effective method of tablet production. However, only a small number of the active pharmaceutical ingredients (APIs) can be successfully manufactured into tablets using DC since most APIs lack adequate functional properties to meet DC requirements. The use of suitable modifiers and appropriate co-processing technologies can provide a promising approach for the preparation of composite particles with high functional properties. The purpose of this review is to provide an overview and classification of different modifiers and their multiple combinations that may improve API tableting properties or prepare composite excipients with appropriate co-processed technology, as well as discuss the corresponding modification mechanism. Moreover, it provides solutions for selecting appropriate modifiers and co-processing technologies to prepare composite particles with improved properties.

## 1. Introduction

The tablet is still the most commonly used solid form due to its dose accuracy, convenience, stability, and ease of production in large quantities [1,2,3,4]. Direct compaction (DC) is the most preferred and is the first choice for tableting [5,6,7]. DC has many advantages over dry and wet granulation, such as eliminating heat and moisture effects, reducing costs and turnaround times, ensuring continuity, simplifying the process, and enhancing bioavailability [8,9,10,11,12].

It was demonstrated that the materials for DC must possess good flowability, excellent compactibility, high bulk density, and low lubricant sensitivity, etc. [5,13,14,15,16,17]. Recent studies have demonstrated that less than 20% of the active pharmaceutical ingredients (APIs) and a small number of commercially available excipients can be effectively manufactured into tablets using DC [8,9,14,15,18]. Moreover, the optimization of powder properties is essential for ensuring a robust tablet manufacturing process [19,20,21]. Therefore, it is important to develop new materials with high-functional properties for DC.

The physical properties of powders can be divided into fundamental properties and functional properties [9,22,23,24]. In our previous studies, we have discussed and demonstrated that (i) the functional properties (e.g., compactibility, flowability, lubricant sensitivity, tabletability, dilution potential, and disintegration time, etc.) are determined by fundamental properties (e.g., particle size, surface area, porosity, and density, etc.), which, in turn, are mainly determined by particle structure (e.g., particle morphology and shape); (ii) these properties are interdependent; and (iii) the particle structure is mainly affected by the preparation technologies of powders [6,9,18]. Therefore, it is reasonable to assume that reasonable modification of the particle structure can improve the functional properties of particles significantly.

A composite particle (CP) is defined as a specific combination of two or more established APIs and/or excipients at a sub-particle (even molecular) level designed to physically modify their properties in a way not achievable by simple physical mixing [19,25,26,27]. In other words, the developing of CPs is a research based on particle engineering, in which two or more types of particles are combined into a single-bodied multifunctional particle with preferred properties, e.g., improved flowability, compactibility, and lubricant sensitivity, etc. [9,28,29]. Moreover, the CPs formed with high functional properties are simply physically modified such that they do not lose their chemical structure and stability. Specifically, the CPs maintain their independent chemical properties, while their functional performance is synergistically increased [8].

Co-processing, a highly effective technique, is often employed to prepare CPs with improved surface modification of particle structure and excellent functional properties [14,17,19,27]. Surface modifiers (e.g., hydroxypropyl methylcellulose (HPMC), polyvinylpyrrolidone (PVP), and silica, etc.) are often utilized to modify the particle structure, thus, improving the properties of materials with poor DC properties [6,28,30,31,32]. Generally, these materials exhibit excellent properties for DC, such as good compactibility and flowability, fast disintegration, and low hygroscopicity. Therefore, surface modifiers are important for DC. Meanwhile, it is also important to reasonably and properly choose surface modifiers for DC, as different surface modifiers have varied characteristics and functions, e.g., HPMC exhibits excellent compactibility, low hygroscopicity, and slow disintegration; PVP exhibits excellent compactibility, high hygroscopicity, and has no effect on disintegration; silica exhibits excellent flowability and poor compactibility; and magnesium stearate (MgSt) prevents sticking, but compactibility is sacrificed [8,15,17,18,33].

According to the published literature, four aspects of CPs are mostly emphasized: (i) exploring and/or comparing the effect of different surface modifiers on CPs; (ii) characterizing the functional properties of CPs; (iii) developing multifunctional materials with enhanced performances and various co-processing methods; and (iv) analyzing the mechanisms of CPs property improvement from the powder level [6,19,25,26,28,34,35,36]. Several excellent reviews were also published in this field [5,9,37,38]. Li and colleagues summarized and discussed the improved functional properties of CP from the view of particle structure and co-processing [6,9,18]; Sharma et al. reviewed the application of dry coating, as well as its effects on the flowability and cohesiveness of pharmaceutical powders [39]. The preparation of materials for DC based on SeDeM was also reviewed [4,40,41,42,43]. All the reviews mainly involved the above aspects (ii), (iii), and (iv) but rarely the first aspect (i). According to published reports, different surface modifiers had significantly different effects on CPs prepared using the same procedures. Therefore, this review is intended to provide an updated overview of the impact of various surface modifiers on CPs as well as the differences between variations of the same surface modifier, based mainly on research published within the last five years.

**Figure 1:**
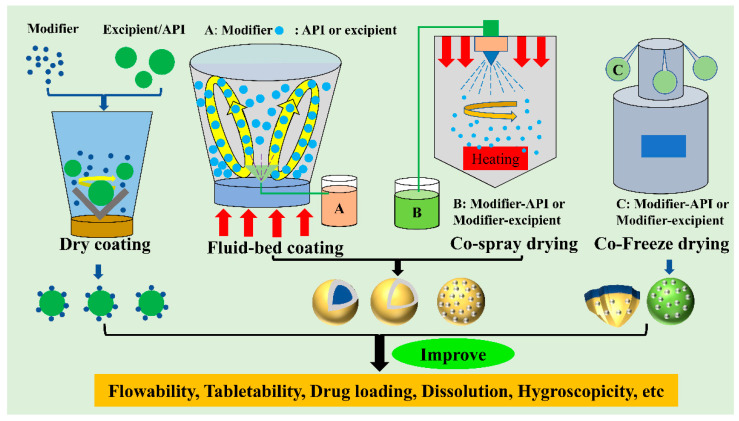
The mechanism of modifiers combined with corresponding co-processing techniques to improve the properties of powders.

Co-processed excipients are commonly considered as the combination of two or more pharmaceutical excipients co-processed by suitable co-processing methods (e.g., spray drying, co-precipitation, and co-crystallization). In co-processing, their properties are improved by physical modification rather than simple physical mixing [44,45]. The development of these co-processed excipients often involved fluid-bed granulation and spray drying, among others [44,46]. In the earlier studies, the aim of co-processed pharmaceutical excipients is to acquire a new composite excipient with better functional properties than the raw excipient [46,47]. The co-processed excipients are also popular in direct compaction since they have excellent key direct compaction properties that could effectively improve the direct compaction properties of drug powders. According to the modification alone or in combination with other surface modifiers, it can be divided into unitary modifier, binary modifier, and ternary modifier [48,49]. According to different co-processed methods, this review summarizes the one-component modifier and multi-component modifier, and discusses the corresponding modification mechanism. Particle properties can be divided into two types: fundamental properties and functional properties. Functional properties, such as flowability, tabletability, hygroscopicity, lubricant sensitivity, wettability, and disintegration time, which are determined by fundamental properties, such as particle size, particle shape, particle morphology, surface area, porosity, and density [9,22,50]. As the fundamental properties of particles are mainly determined by particle structure, the composite particles with high functional properties can be obtained via modifying their fundamental properties [51,52,53,54,55]. This goal can be achieved by adopting suitable modifiers in combination with corresponding modification techniques (Figure 1).

## 2. HPMC (Hydroxypropyl Methylcellulose)

HPMC, a commonly used binder in tablets and a popular material in film coating, is made of alkali cellulose, propylene oxide, and alkane chloride, exhibiting a white or slightly yellow powder state with a tasteless, odorless, and non-toxic nature [30]. HPMC is widely used in the pharmaceutical field for its excellent properties, such as low hygroscopicity (about 10% equilibrium moisture at 75% relative humidity), high glass transition temperature (170–180 °C), and different grades of viscosities [9,56]. In the recent years, HPMC has been primarily used for the following purposes: (i) as the matrix in sustained-release tablets; (ii) water-soluble film material; (iii) capsule shell material; (iv) gel thickener; and (v) powder modifier [57,58,59,60,61].

Since the solution of HPMC has a good viscosity and surface tension, it is broadly used to prepare core-shell composite particles by fluid-bed coating and spray drying technology. The composite particles can effectively improve the tableting properties of drug powders. HPMC can be combined with other modifiers, such as porous mannitol, porous lactose, and ammonium bicarbonate (NH_4_HCO_3_), to form binary and ternary modifiers in order to improve multiple properties of the target drug powder or prepare functional composite excipients.

### 2.1. Unitary Modifier

#### 2.1.1. Co-Spray Drying

In the report from Al-Zoubi et al., metformin hydrochloride (MH), a representative of high dose drugs with poor compactibility, failed to be compacted into intact tablets during the compaction range of 74~444 MPa. Therefore, aqueous feed solutions of MH and HPMC E3 in ratios of 97.5:2.5 or 95:5 were spray dried. When compared to MH, both co-processed products showed improved compactibility: (i) the tablet tensile strength at a porosity of 0.15 increased from 0 MPa to 1.89~2.67 MPa; (ii) the work of compaction, which is related to the ability of a material to absorb work during compression, increased by 66.2~84.4%; (iii) the yield pressure and elastic recovery decreased by 4.5~6.2% and 7.6~21.2%, and the lower yield pressure and elastic recovery generally indicated plastic deformation and enhanced compactibility [28]. This could be attributed to the following aspects: (i) HPMC E3 inhibited the crystal growth of MH during co-spray drying, thus, the relative crystallinity of co-processed products reduced by 3.5~6.8%. The amorphous materials are more prone to plastic deformation and enhanced compactibility than crystal material; (ii) co-processed products show spheroidal particle structure, while MH contains large prismatic particles, which hinders compactibility; (iii) HPMC E3 formed a relatively intact shell layer on the surface of MH particles, forming a core-shell CP; and (iv) HPMC E3 showed much better compactibility than MH.

Similar studies were also reported for the preparation of functional excipients, such as starch, anhydrous dibasic calcium phosphate (DCPA), and mannitol as three types of commonly used tablet fillers [62]. They are not suitable for the direct compaction process due to poor flowability and tabletability. Therefore, HPMC was chosen as a surface modifier to improve their compaction properties by co-spray drying. Aqueous feed solutions of starch or DCPA, or mannitol with 7% HPMC E3 were co-spray dried to prepare composite products under the condition of inlet temperature 180 °C and feed rate 22 of mL/min. When compared with the unprocessed particles, three composite products exhibited improved flowability and tabletability: (i) the angle of repose (AR) reduced from 48.3°, 52.7°, and 54.3° to 46.8°, 37.5°, and 34.6°, respectively; (ii) the Hausner ratio reduced from 1.73, 1.99, and 1.67 to 1.52, 1.50, and 1.49, respectively; (iii) the tableting ratio decreased by 6.28%, 23.03%, and 2.32%, respectively; and (iv) the yield pressure decreased by 23.24%, 26.34%, and 24.69%, respectively. Lower yield pressure indicated enhanced plastic deformation and compactibility; and (v) the tablet tensile strength increased by 4.00-fold, 4.50-fold, and 2.01-fold, respectively. The improvement mechanism could be summarized as follows: (i) the particles prepared by co-spray drying with HPMC showed an increase in particle size, which led to improve flowability; (ii) the shape of composite particles was spherical, which contributed to improved flowability; and (iii) the HPMC E3 as the shell material was homogenously distributed on the surface of the primary particle, which resulted in better plastic deformation. Similar results were reported by other researchers [19,63].

#### 2.1.2. Co-Freeze Drying

Co-freeze drying includes three main steps, freezing, primary drying, and secondary drying. It is widely used in the manufacturing and pharmaceutical fields [64]. Furthermore, it was previously investigated as a novel secondary processing technique for preparing co-processed cushioning excipients with a porous and fluffy structure [50,65].

Co-processed excipients also had widespread applications in multiple-unit pellet systems (MUPS) [66,67]. However, producing MUPS tablets requires cushioning excipients to protect the pellet coating under the compaction force. Generally, cushioning excipients have the following features: protecting the coated pellets, preventing the fusion of tablets during compaction, accomplishing the minimum segregation under the compaction, and offering good flow properties. In the report from Carin Ru et al., mannitol was selected as a model material to prepare a cushioning excipient [64]. The ratio of mannitol to HPMC and HPMC viscosity grades were the key factors in determining the quality of cushioning excipients prepared by the co-freeze-dried process. When compared with the tablets without appending cushion excipients, the co-freeze-dried mannitol–HPMC (F4M) (*w*/*w*, 3:1) excipients exhibited the best cushioning performance, excellent tabletability, and better dissolution. First, the disintegration time decreased by 41%, and second, the tablet tensile strength increased by 2.50~5.20-fold. These could be attributed to the following aspects: (i) porous mannitol has disintegration-enhancing effects [6,30], which mitigated the slow disintegration caused by the gel-forming nature of HPMC; and (ii) the fragmentation of co-freeze-dried cushioning excipients provides new surface for bonding, resulting in increasing bonding area. The larger bonding area would enhance interparticle bonds, thereby increasing the tablet’s tensile strength.

#### 2.1.3. Fluid-Bed Coating

Fluid-bed coating is widely used in pharmaceutical industry to physically modify powders to obtain targeted particles, e.g., masking unpleasant tastes and improving functional properties of APIs [6,68,69,70]. In recent years, some researchers employed fluid-bed coating as a path to prepare core-shell particles for DC. In the report from Li Z et al., *Zingiberis rhizoma* extracted powder (ZR) was chosen as the model drug that had poor flowability and tabletability, and HPMC E3 was selected as the coating material [6]. The mass ratio of the HPMC to ZR was 7:93, and the concentration of HPMC aqueous solution was 13%. In comparison to raw particles, the composite particles containing ZR and HPMC showed improved DC properties: (i) AR was reduced from 46.60° to 31.02°; (ii) the Hausner ratio was reduced from 1.41 to 1.30; (iii) the Carr’s index was decreased from 29.2 to 23.2; (iv) the area under tensile strength vs. compaction force curve was increased from 0 MPa·kN to 8.79 MPa·kN; and (v) the equilibrium hygroscopic moisture content decreased by 16.94%. All of these results indicated that the flowability, tabletability, and hygroscopicity were improved. This could be ascribed to the following aspects: (i) the core-shell composite particles had the bigger particle size and better size uniformity. The median particle size (d _(0.5)_) of composite particles was 3.95-fold larger than parent ZR. The uniformity of composite particles was decreased from 2.3 to 1.7; (ii) HPMC coated on the surface of raw ZR particles formed spherical agglomerates, increasing the distance and decreasing the contact area of the cohesive ZR particles; (iii) HPMC as the shell layer had the better bonding capability and plastic deformation; (iv) the core-shell composite particles exhibited a 250.80-fold higher hardness and 22.27-fold larger cohesiveness, which were beneficial for tabletability [5,6,71,72,73]; (v) the solid and liquid bridges formed during the fluid-bed coating were also conducive to improving the compactibility, (vi) the composite showed better fragmentation than raw materials. The composite materials often show fluffy agglomerates, and such agglomerates are broken and redistributed into small particles, leading to increased interparticle bonding area and intermolecular bonding, thus, showing excellent tableting properties. A similar approach was also successfully applied to improve the DC properties of calcium carbonate and mannitol [30].

#### 2.1.4. Co-Milling

Co-milling is defined as the milling of an excipient/API in the presence of another excipient or multiple excipients/APIs. Milling and co-milling are well known techniques in the pharmaceutical industry, having a positive influence on the kinetic solubility and dissolution rate of sparingly soluble drugs [74,75,76]. The milling of drug reduces the drug particles to the micron or submicron level and provides a larger surface area, which results in the improvement of wettability and dissolution [77,78,79]. However, the milling of powder also generates certain shortcomings, e.g., the coarser particles are turned into finer ones that are more energetic and therefore result in a higher surface energy. This increases the interactions among particles, increases their cohesiveness, and increases the aggregate size, which contributes to poor dissolution [53,80]. In order to overcome these disadvantages and improve the milling efficiency, a favorable technique by co-milling with appropriate surface modifiers was successfully applied [77,79,81,82]. Co-milling or co-grinding of drugs with various surface modifiers, such as lactose, MCC, starch, PVP, HPMC, etc. is a promising approach to improve their wettability, flowability, and dissolution [83,84,85].

In a recent study reported by Amjad and coworkers, ibuprofen was chosen as the model drug, which is practically insoluble in an aqueous or acidic medium [74]. Ibuprofen cannot be milled alone as it is a highly ductile material with a low melting point [86]. HPMC was selected as the unitary modifier. The ratio of ibuprofen to HPMC was 1:0.5. They were co-milled by ball-milling equipment at a speed of 18 Hz and for a time of 15 min. In comparison with the pure drug, ibuprofen co-milled with HPMC exhibited higher solubility (0.53 mg/mL vs. 0.09 mg/mL) and showed a better dissolution rate, as the time decreased from 72 min to 20 min when the cumulative drug release was 70%. The reason for these improvements can be attributed to the following three aspects. First, the reduction in particle size and the amorphization of crystalline substance, as the reduction in the particle size would be accompanied by a dramatic increase in the surface area of composite particles, hence, exhibiting better dissolution behavior. Second, HPMC coated on the surface of ibuprofen particles could significantly weaken the crystallinity of the co-products. Third, the addition of HPMC could stabilize the amorphous phase and increase the solid-state hydrogen bonding. Additionally, HPMC is widely used as the unitary modifier to improve the dissolution ability of other drugs by co-milling [87,88,89,90].

#### 2.1.5. Crystallo-Co-Agglomeration

Crystallo-co-agglomeration (CCA) is a novel particle-engineering technique that adopts appropriate modifiers and suitable solvents for co-processing with the drug, producing spherical crystal products with improved micromeritic and mechanical properties, solubility, and dissolution [91,92,93]. CCA is a modification of spherical crystallization involving the use of a bridging liquid to form agglomerates of the target drug and modifier. In the process of CCA, a drug is dissolved in a solvent, and a modifier is dispersed in the drug solution. Then, a bridging liquid is inserted as an agglomerating agent, resulting in crystallization and agglomeration.

In the paper by Rosenbaum T et al., an API (a commercial confidential compound manufactured by Bristol Myers Squibb) was selected as the model drug, dissolved in water, and HPMC was add as a modifier to the API solution. They were co-processed by CCA, and the ratio of API to HPMC was 3:1 [94]. Compared to the pure API, the co-processed product showed improved flow properties and sustained release behavior. The bulk density was increased from 0.14 g/mL to 0.34 g/mL, and the value measured by the Erweka flow test was improved from no flow to 4.13 g/s. This might be attributed to the swelling of the polymer in water, allowing the API to be absorbed along with water into the HPMC matrix, thereby increasing the particle size.

### 2.2. Binary Modifiers

In the report from Wang ST et al., lactose (pharmatose 90 M) was chosen as the core material, and HPMC E3 and cross-linked polyvinylpolypyrrolidone (PVPP) were selected as the shell materials. They were co-spray dried to prepare a novel ternary composite excipient. The ratio of lactose to HPMC and PVPP was 89.5:7:3.5 [95]. Compared to the unitary lactose, the ternary modifier showed improved flowability and tabletability: (i) the AR and Hausner ratio decreased from 48.7° to 45.0° and 1.67 to 1.57, respectively; and (ii) the compaction ratio decreased from 22.81% to 20.00%; (iii) the yield pressure decreased from 179.7 MPa to 113.0 MPa. The mechanism of improvement can be summarized in the following ways: (i) the HPMC and PVPP was distributed on the surface of the lactose, which could improve the binding properties and plastic deformation nature; and (ii) the composite particles showed significant changes in the shape and surface topography. The unprocessed particles showed an irregular shape and density, but ternary particles showed a spherical and porous nature, which could provide a bigger surface area, thereby enhancing the compactibility of co-processed excipients. The applications of HPMC were summarized in Table 1 [5,6,18,28,30,44,62,64,96,97,98,99,100].

## 3. PVP (Polyvinylpyrrolidone)

Polyvinylpyrrolidone (PVP) is a water-soluble polymer formed by the polymerization of N-vinyl-pyrrolidone [101]. In the absence of water, it exhibits a white powder or granular state. The K value of PVP is usually used to represent different sizes and viscosity levels, and the range of the K value is generally between 10 to 100 [102,103]. The larger the K value, the larger the molecular weight. PVP as a common pharmaceutical excipient has broad applications, such as a tablet binder, solid dispersion carrier, pore-former in sustained and controlled release preparation, and cosolvent in insoluble drugs [101,104,105]. In recent years, with the development of particle engineering, PVP is commonly used in particle design to prepare composite particles by fluid-bed coating, spray drying, and freeze drying [106,107,108].

### 3.1. Unitary Modifier

#### 3.1.1. Co-Spray Drying

In the study by Sadeghi F et al., acetaminophen, a representative drug with high crystallinity, failed to be compacted into intact tablets by direct compression. Therefore, aqueous feed solutions of acetaminophen and PVP K30 in ratios of 95:5 were co-spray dried [108]. When compared to untreated acetaminophen, the co-processed products showed improved dissolution and excellent compaction properties: (i) the mean dissolution time decreased from 19.7 min to 2.8 min; (ii) the crushing strength of tablets was increased from 10 N, 20 N, 18 N to 70 N, 105 N, 110 N under the compaction force of 10 kN, 15 kN and 20 kN, respectively; and (iii) the percent crystallinity was decreased from 100% to 36.31%. The mechanism of improvement in the tablet properties can be described as a variation in particle morphology, a decrease in crystallinity, and an increase in particle size. The particle morphology was changed from an acicular or rod shape to spherical, which was beneficial in improving the tabletability of composite particles. Generally, amorphous materials have better dissolution and compactibility than crystalline materials due to the restriction of the crystal lattice energy of crystalline [109,110]. PVP as a strong crystal growth inhibitor in acetaminophen may improve the percentage of amorphous components in composite materials. The PVP distributed on the surface of acetaminophen particles could induce the adhesion of particles to each other and facilitate agglomerate formation, thereby enhancing the crushing strength of tablets [111,112]. Additionally, PVP is a water-soluble polymer whose dispersion on the surface of primary particles may enhance the wettability of the integral particles, thereby improving dissolution and compactibility.

In another similar study reported by Al-Zoubi N and coworkers, metformin hydrochloride (MH), a representative of high dose drugs with poor compactibility, failed to be compacted into intact tablets during the compaction range of 74~444 MPa. Therefore, aqueous feed solutions of MH and PVP K30 in ratios of 95:5 were co-spray dried. When compared to MH, the co-processed products showed improved compactibility: (i) the tablet tensile strength at a porosity of 0.15 increased from 0 to 2.00 MPa; (ii) the work of compaction, which relates to the ability of a material to absorb work during compression, increased by 72.87%; (iii) the yield pressure and elastic recovery decreased by 19.23%; the lower yield pressure and elastic recovery are generally associated with enhanced plastic deformation and compactibility [28]. This could be attributed to the following aspects: (i) PVP K30 inhibited the crystal growth of MH during co-spray drying, thus, the relative crystallinity of co-processed products reduced by 10.00%. The amorphous material is more prone to plastic deformation and enhanced compactibility than crystal material; (ii) co-processed products showed a spheroidal particle structure, while MH still has large prismatic particles, which reduced compactibility; and (iii) PVP K30 showed much better compactibility than MH and formed a relatively intact shell layer on the surface of MH particles, forming a core-shell CP.

In addition, PVP can be used as the templating agent to prepare porous particles. In a study reported by Zhu WF et al., the PVP K30 was utilized as the templating agent to prepare porous lactose, which was successfully applied to improve the dissolution behavior of curcumin. The ratio of lactose to PVP was 99:1, 98:2, and 97:3, respectively. First, they were separately prepared by co-spray drying. Then, the PVP was removed to prepare porous lactose [113]. When compared to the unprocessed lactose, the co-processed products exhibited improved flowability: (i) the AR was decreased from 56.7° to 42.2°, 40.5°, 40.0°, respectively; (ii) the Carr’s index was decreased from 47.0 to 31.7, 28.7, 32.3, respectively; and (iii) the Hausner ratio was decreased from 1.89 to 1.46, 1.40, 1.48, respectively. The curcumin was utilized as the model drug to investigate the dissolution effects of the porous lactose. The results showed that the dissolution percentage of cumulative release was increased from 23% to 86%, 98%, 93%, respectively. All of the above could be due to the spheroidal porous structure and improved amorphous material properties.

#### 3.1.2. Co-Freeze Drying

In a recent work reported by Volkova TV et al., leflunomide was co-freeze dried with PVP to improve dissolution [114]. The results showed that compared to unprocessed leflunomide, the co-processed product dissolution efficiency (%) of leflunomide was increased in 10, 60, and 120 min from 0–1.05% to 10.73%, 61.46%, and 78.44%, respectively. The increase in the dissolution rate of leflunomide might be due to the following three reasons: (i) partial loss of drug crystallinity. The crystal state turned into the amorphous state after co-freeze drying; (ii) the increase in surface area due to the fact that the co-processed drug exhibits solid dispersion, which increases the area of contact between the drug and dissolution medium; and (iii) the incorporation of PVP as a hydrophilic carrier, which improved the wettability of the drug.

#### 3.1.3. Fluid-Bed Coating

In a report conducted by Li Z et al., PVP K30 was selected as the unitary modifier to improve the flowability and tabletability of *Zingiberis rhizoma* extracted powder (ZR), which was unable to be compacted into tablets directly due to the poor compactibility and flowability. The ratio of ZR to PVP K30 was 93:7, which were then co-processed by a laboratory fluid-bed coating machine [6]. Compared with the unprocessed raw powder, the composite products showed improved flowability, compactibility, and hygroscopicity. First, the values of AR, Hausner ratio, and Carr’s index decreased from 46.6°, 1.4, and 29.2 to 30.7°, 1.3, and 22.3, respectively. Second, the area under the tensile vs. compaction force curve increased from 0 MPa·kN to 9.279 MPa·kN. Third, the equilibrium hygroscopic moisture content decreased from 18.2% to 15.5%. Similar results were found in another study [97], where three ethyl-alcohol-extracted powders (*Poria*, *Puerariae Lobatae Radix*, *Zingiberis rhizoma*), three water-extracted powders (*Andrographis herba*, *Ganoderma*, *Gardeniae fructus*), and three directly pulverized powders (*Citri Reticulatae Pericarpium*, *Andrographis herba*, *Poria*) were chosen as the model drugs with poor flowability and tabletability, and PVP was employed as the modifier. They were co-processed by fluid-bed coating.

The mechanism of the modification mentioned above could be summarized into two parts. First, it improved the particle structure and fundamental properties, e.g., spheroidal shape and smoother surface, increased particle size, more uniform size distribution, lower bulk, and tapped densities, which were conducive to the functional properties. Second, the shell material could increase the distance and decrease the contact of the cohesive raw particles, which leads to the reduction in van der Waals forces between composite products. Moreover, the enhanced compactibility could also be the result of excellent plastic deformation of shell materials, and the phenomenon appeared in the process of forming core-shell particles, e.g., the particle–particle collisions, solid and liquid bridge formation, and interlocking between particles.

### 3.2. Binary Modifiers

#### 3.2.1. Co-Spray Drying

In the research reported by Vanhoorne V et al., paracetamol was used as a model drug that had poor flowability and a high tendency for capping during compaction. PVP and lactose were selected as the surface modifiers to improve the properties of paracetamol. The ratio of paracetamol, PVP, and lactose was 75:5:20, respectively. The composite products of the three materials were prepared by co-spray drying [115]. Compared to the unprocessed paracetamol particles, the composite particles showed improved tabletability and flowability. The flowability index, which is an indicator of flowability and positively related to the flowability, increased from 1.3 to 2.9. The tensile strength was increased by 7.4 times. All the above results could be attributed to the following reasons. The use of PVP as a crystallization inhibitor may enhance tabletability by increasing the amount of the amorphous form in the products during co-spray drying. Lactose is a low molecular sugar that could reduce the glass transition temperature, thus, possibly improving cohesion between composite particles. Both PVP and lactose mainly displayed plastic behavior during tableting. Furthermore, the binder–binder interaction between two modifiers could make some elastic recovery of paracetamol without breaking the inter-particulate bonds during compaction. The improved surface morphology and increased particle size of composite particles were also conducive to the tabletability and flowability.

#### 3.2.2. Fluid-Bed Coating

In a report studied by Li Z et al., PVP K30 not only acted as the unitary modifier, but could also serve as a binary modifier with mannitol. The ratio of *Zingiberis rhizoma* to PVP and mannitol was 93:1.4:5.6, respectively, and the composite particles were produced by fluid-bed coating [6]. Compared to the raw *Zingiberis rhizoma*, the AR, Hausner ratio, and Carr’s index of composite particles decreased from 46.60°, 1.4, and 29.2 to 30.11°, 1.3, and 22.4, respectively; and the area under the tensile vs. compaction force curve of composite particles increased from 0 MPa·kN to 5.498 MPa·kN. The mechanism of modification was similar to that concluded in Section 3.1.3. Compared to corresponding unitary modifier (PVP or mannitol), the binary modifier showed different improvements in properties, which were mainly attributed to their differences in viscosity and surface tension.

#### 3.2.3. Dry Coating

In the research reported by Qu L et al., a binary modifier containing PVP and magnesium stearate was used to improve the tensile strength of the ibuprofen tablets. The ratio of ibuprofen to PVP and magnesium stearate was 89:10:1, respectively. The composite powder was prepared by dry coating [36]. Compared to the unprocessed powder, the tensile strength of co-processed products increased by 77% (0.96 MPa vs. 1.70 MPa). This could be attributed to the high plastic deformation characteristics of PVP during compaction and the formation of core-shell structure composite particles. The applications of PVP were summarized in Table 2 [6,28,36,97,108,113,114,115,116,117,118,119,120,121,122,123].

## 4. SiO_2_

Silica (SiO_2_), a form of silicate that contains silicon and oxygen, is abundant on earth and can be found in a variety of forms. It needs to be processed or manufactured using synthetic chemical processes before being used in pharmaceutical and other fields [124,125,126,127]. Due to the high surface area, excellent biocompatibility, adjustable surface area, porous structures, and excellent functionality, nano- and micro-sized silica have certainly attracted the attention of many researchers in the medical fields [128,129]. As per surface polarity, silica can be classified into hydrophilic and hydrophobic groups [130]. Silica can play multiple positive roles in solid preparations, such as improving compactibility, flowability, and wettability of powders, reducing the sticking and capping effect during tableting, and promoting tablet disintegration [131].

Due to its small size, silica has a large specific surface area (about 100–400 m^2^/g depending on grade) [132,133]. Thus, silica has abundant surface free energy and has the ability to physically adsorb onto the surface of surrounding materials. It is widely used as the texture or surface modifier to modify surface properties of drug particles by dry or wet coating and liquid dispersion methods. A smaller amount of silica as a modifier can effectively improve the compactibility, disintegration, hygroscopicity, flowability, adhesion, and powder packing of host particles [134,135,136,137].

### 4.1. Unitary Modifier

#### 4.1.1. Dry Coating

Dry coating is widely regarded as a promising method to substantially improve the flowability, dispersibility, and hygroscopicity of cohesive powders in the pharmaceutical field [25,135]. During the dry coating process, a layer of nano-sized particles (guest particles), e.g., nano-silica, is usually employed to coat the surface of a larger particle (host particle) through mechanical forces. Generally, the host particle can be the API or excipient. During typical pharmaceutical blending, silica usually does not de-agglomerate sufficiently, resulting in uneven coating on the drug powders. In contrast, dry coating by mechanical forces can make the nano-silica distribute on the surface of host particles more uniformly. Because it can decrease the natural surface roughness of the host particles, thereby, it reduces the cohesion between the host particles and leads to smaller Bond number values [25,34,138,139,140].

In the paper reported by Kunnath K et al., three cohesive APIs, such as micronized acetaminophen, coarse acetaminophen, and micronized ibuprofen, were chosen as the model drugs. They were dry coated with 1% nano-silica (hydrophobic or hydrophilic) to improve their flowability and tabletability [135]. Compared to the uncoated raw drugs, the co-processed products showed improved flowability and tableting properties. First, the flow function coefficient values of the co-processed products being coating with hydrophobic silica increased from 3.4, 1.9, 2.3 to 13.8, 8.4, 5.8, respectively. The bigger the value of the flow function coefficient was, the better the flowability of the powders was. Second, the tensile strength of the co-processed products coated with the hydrophobic silica increased from 1.4 MPa, 0.8 MPa, 1.9 MPa to 1.7 MPa, 1.1 MPa, 2.3 MPa, respectively. The co-processed products coated with hydrophilic silica also demonstrated similar results. The modification mechanisms could be due to the reduction in inter-particle cohesion, increase in the bulk density and total surface area after coating with nano-silica. Moreover, the presence of nano-silica also facilitated the deagglomeration and rearrangement of particles during compaction.

In another similar piece of research published by Huang ZH and colleagues, micronized acetaminophen, α-lactose monohydrate Pharmatose 450, and microcrystalline cellulose Avicel PH-105 were selected as the host materials. They were co-processed with nano-silica R972P to improve their direct compaction properties by dry coating [140]. The weight percentage of guest particles to micronized acetaminophen, Pharmatose 450, and Avicel PH-105 was 2.68%, 0.99%, and 1.97%, respectively. Compared to the unprocessed primary materials, the co-processed products exhibited improved flowability and tabletability: the flow function coefficient, a positive correlation indicator of flowability, increased to 229%, 279%, and 522%, respectively; the bulk density increased to 210%, 154%, and 134%, respectively, thus, showing improved fallibility for tableting; and the tensile strength was also increased from less than 2.00 MPa to more than 2.00 MPa at a drug loading of 60%. The improvement mechanism was similar with the discussion summarized in the previous paragraph.

In other similar research, microcrystalline cellulose PH102 was dry coated with nano-silica to prepare a composite excipient in order to improve the direct compaction properties of acetaminophen [34]. The weight percentages of PH102 and nano-silica were 10.1% and 1.0%, respectively. The co-products showed excellent flowability and tabletability. The corresponding flow function coefficient and tensile strength improved separately by 3.02 times, 4.30 times, 5.00 times and 1.24 times, 1.08 times, 1.33 times, respectively, at drug loadings of 20%, 40%, and 60%. Accordingly, the modification mechanism was the combination of the two excipient characteristics. First, fine-sized microcrystalline cellulose PH102 could be advantageous to increase the bonding area during compaction, which improved interparticle bonds, including solid bridges, intermolecular forces, and mechanical interlocking. Second, nano-silica coated on the surface of primary particles could significantly improve the flowability of composite products [35,141].

#### 4.1.2. Liquid Dispersion

Liquid dispersion is a method of mixing liquids with each other, which could cause the modifier to evenly distribute within and/or on API particles for modifying their surface and texture. It was considered to be a suitable surface modification method, especially for the liquid extracts of traditional Chinese medicine and chemical drug solutions [26].

In a recent published study, colloidal silica N20 was used as the unitary surface modifier to improve direct compaction properties of *Zingiberis rhizoma* extracted powder (ZR) by liquid dispersion [133]. The ratio of ZR to nano-silica separately was 1:0.25, 1:0.33, 1:0.41, and 1:0.5. Compared with the unprocessed ZR, the nano-silica-modified composite particles exhibited lower values of AR (46.6° vs. 38.1°, 38.0°, 37.6°, 36.9°), Carr’s index (35.49 vs. 30.96, 29.84, 25.91, 23.65), Hausner ratio (1.55 vs. 1.45, 1.43, 1.35, 1.31), powder flow time (unable to flow vs. 30.3 s, 25.0 s, 23.7 s, 8.0 s) and hygroscopicity (5.15% vs. 4.58%, 3.94%, 3.56%, 3.41%), and higher values of bulk density (0.64 g/cm^3^ vs. 0.79 g/cm^3^, 0.80 g/cm^3^, 0.79 g/cm^3^, 0.75 g/cm^3^), hardness (15,749 g vs. 40,682 g, 46,369 g, 52,744 g, 56,712 g), cohesiveness (0.31 vs. 0.64, 0.67, 0.73, 0.83), and tensile strength (1.5 MPa vs. 3.3 MPa, 3.5 MPa, 5.5 MPa, 7.0 MPa). All of these demonstrated that the composite particles showed improved flow properties and tablet-forming properties. The possible modification mechanisms could be concluded as follows: (i) the structure of composite particles showed a nearly spherical shape, which improved the flowability; (ii) the coating material effectively and uniformly distributed on the surface of ZR particles, which, as a result, could reduce contact area and increase inter-particle distance between cohesive primary ZR particles, hence, decreasing the van der Waals force; (iii) the distribution of the silica on the composite particle surface could form a sandwich with the two adjacent composite particles, which results in the formation of solid and liquid bridges; and (iv) larger hardness and cohesion, which are often used to characterize the particle strength and bonding strength of material [6].Composite particles showed improved hardness and cohesion, thus, exhibiting improved tableting and more easy to form, qualified tablets.

In another similar research reported by Gao YT et al., the *Citri Reticulate Percarpium* powder (CB) was co-processed with hydrophilic or hydrophobic nano-silica by liquid dispersion in order to improve the direct compaction properties [26]. The ratios of CB to hydrophilic and/or hydrophobic nano-silica were 16.7:1 and 10:1, respectively. When compared to CB, the main changes in properties of the co-processed products based on hydrophilic nano-silica are summarized as follows: the bulk density increased from 0.456 g/mL to 0.590 g/mL and 0.639 g/mL, respectively; the AR decreased from 35.58° to 31.62° and 33.78°, respectively; the Carr’s index and Hausner ratio showed some improvement; the tensile strength increased from 0.3 MPa to 0.5 MPa and 2.3 MPa, respectively. While, in comparison to CB, the main changes in the properties of the co-processed products based on hydrophobic nano-silica were summarized as follows: the bulk density was separately increased from 0.456 g/mL to 0.557 g/mL and 0.656 g/mL, respectively; the AR decreased from 35.58° to 27.82° and 33.53°, respectively; the Carr’s index decreased from 36.67 to 31.33 and 32.33, respectively; the Hausner ratio decreased from 1.58 to 1.46 and 1.48, respectively; and the tensile strength increased from 0.3 MPa to 1.2 MPa and 0.5 MPa, respectively. Furthermore, the corresponding tablets based on the co-processed products also exhibited suitable disintegration time and improved drug loading. All tablets completely disintegrated within 7 min. Therefore, the tablets prepared with the co-processed products containing CB and hydrophilic nano-silica (10:1) had qualified tensile strength 2.3 MPa at a drug loading of 91%. The modification mechanism of nano-silica, on the whole, was similar to what was discussed in the last paragraph. Additionally, nano-silica has a nano-scaled primary particle size and hard texture, often exhibiting little plastic deformation during compaction, but it can effectively increase the bonding area between particles, thus, leading to the same effect on plastic material. Finally, the nano-silica coating around the host particles hindered the moisture, thus, reducing the cohesive effect induced by dampness. Except for what was mentioned above, the results also demonstrated that there were different modification effects between hydrophilic and hydrophobic nano-silica. This could be explained by their different distribution in primary particles and different efficiency in weakening intermolecular forces. In general, hydrophobic nano-silica had a weaker affinity for raw particles, thus, showing more suitability for coating the surface of CB [26].

#### 4.1.3. Co-Milling

In the research reported by Mullarney MP et al., ibuprofen, a cohesive with poor flowability, was selected as the model drug. One percent (*w*/*w*) silicon dioxide (Aerosil 200) was chosen as the unitary surface modifier. They were co-processed by co-milling technology to improve the flowability and bulk density of ibuprofen particles [51]. The co-milling conditions: the impeller tip speed was 2.4 m/s, the feed rate was 1 to 10 kg/h, and the screen hole sizes were 0.018, 0.024, and 0.032 inch. In this process, silicon dioxide was dispersed onto the surface of host particles (ibuprofen particles) forming the composite particles with the special core-shell structure. Compared to the pure ibuprofen particles, the co-processed products exhibited a considerable improvement in flow performance and bulk density (flow function coefficient value: 3.0 vs. >10.0; bulk density: 0.42 g/cm^3^ vs. 0.50–0.56 g/cm^3^). The modification mechanisms could be summarized by the following two points: First, the silicon dioxide particles with well-dispersing properties distributed on the surface of ibuprofen particles, which could effectively increase the distance and decrease the contact area of separately coated cohesive host powders, and thus, reduces the van der Waals forces between composite particles and ultimately exhibit better flowability. Second, the silicon dioxide particles inhibit the cohesive attractions among host particles, which allows them to rearrange more easily, ultimately increasing the bulk density of composite particles.

### 4.2. Binary Modifiers

Li Z et al. revealed that the binary modifier of the combination of HPMC and silica has more advantages in improving the DC properties of *Zingiberis rhizoma* extracted powder (ZR), when compared with the unitary modifier of HPMC [6]. In their study, *Zingiberis rhizoma* extracted powders, a representative of traditional Chinese medicine materials, which cannot be compacted into tablets directly due to quite poor compactibility and flowability. HPMC and the combination of HPMC and silica were used as the coating excipients. The mass ratio of drug to modifier were 93:7 and 92:7:1, respectively. They were co-processed by fluid-bed coating. Comparing to the raw powder, the co-products formed a core-shell structure and exhibited improved DC properties via fluid-bed coating. First, the composite particles exhibited significantly (ANOVA, *p* < 0.001) improved flowability (unitary modifier: AR: 31.02°, CI: 23.29, HR: 1.30; binary modifiers: AR: 30.23°, CI: 22.43, HR: 1.29) compared to parent ZR (AR:46.60°, CI: 29.02, HR: 1.41). Second, the composite particles also showed improved compactibility. The tensile strength under the compaction force of 10 kN of the parent ZR, composite particles with unitary modifier and composite particles with binary modifiers were 0 MPa, 2.1 MPa, and 3.2 MPa, respectively. Furthermore, the area under the tensile strength vs. compaction force (2~10 kN) curves were 0 MPa·kN, 8.79 MPa·kN, and 14.40 MPa·kN, respectively. Third, the equilibrium hygroscopic moisture content was 18.21%, 15.12% and 14.42%, respectively, which indicated that the composite particles exhibited a significantly lower hygroscopicity. All of the above demonstrated that silica can further improve the functional properties of ZR. Silica acting as an isolation layer could effectively improve the defective surface properties of ZR, such as hygroscopicity and cohesiveness, thus, reducing agglomerations. Additionally, silica adsorbed onto the surface of a composite particle could further improve the flowability by reducing inter-particulate friction. Furthermore, the binary coating liquid exhibited higher viscosity (195.70 mPa·s vs. 178.90 mPa·s) and lower surface tension (39.70 mN/m vs. 40.30 mN/m) than unitary liquid. The higher viscosity and lower surface tension of the modifier liquid were conducive to enhance the bonding force of materials and contributed to modifier spread ability and wettability on the surface of core materials, thus, forming a more perfect core-shell structure. The applications of SiO_2_ were summarized in Table 3 [14,25,33,34,35,51,53,133,139,140,141,142,143,144,145,146,147,148,149,150,151].

## 5. MCC

Microcrystalline cellulose (MCC) is a commonly used dry binder that is obtained from cellulose and was first discovered by Battista and Smith in 1955 [152,153]. MCC, as a frequently used tablet excipient, is a green, renewable, cost-effective, and biocompatible polymer [154]. MCC is one of the most popular cellulose derivatives and is well known in the pharmaceutical industry for its outstanding tabletability. MCC, as an ideal diluent and dry binder, is usually included in DC tablet formulations containing APIs with poor tabletability in order to achieve desirable tablet quality. MCC is also used as a surface modifier for improving the DC properties of brittle materials by co-processing technology due to its excellent plastic deformation ability, rod-shaped particle morphology, high surface area, and outstanding particle dispersibility [34,155,156,157].

### 5.1. Unitary Modifier

#### 5.1.1. Co-Spray Drying

In a study, mannitol, a water-soluble excipient, was selected as the model material that had poor tableting and flow properties, leading to poor direct compaction [158]. MCC was used as the modifier to improve its DC properties by co-spray drying. The mass ratio of mannitol to MCC was 1.25:1, respectively. Compared to the raw mannitol, the co-spray dried powder showed better flowability and tabletability. The hardness of tablets was increased from <1 kg/cm^2^ to 9.8 kg/cm^2^, and the AR was decreased from not being able to be measured to 31.4°. These improvements could be attributed to the following two factors. First, the composite particles display a spherical shape and smooth surface, which are crucial for better flowability. Second, MCC exhibits excellent plastic deformation characteristics and distributes well on the surface of mannitol particles, which can significantly enhance the tableting properties of integrated particles.

#### 5.1.2. Liquid Dispersion

In a recent work by Zhang Y et al., *Puerariae Lobatae Radix* was selected as the model drug, and MCC PH101 was chosen as the modifier. The liquid dispersion method was used to prepare composite particles with 1:0.06, 1:0.25, and 1:0.5 (***w***/***w***) of the model drug with a modifier of high, medium, and low drug loading, respectively [159]. Compared to unprocessed drug powders, the co-processed products in three proportions showed improved key DC properties: (i) the AR was decreased from 52.1° to 46.8°, 47.4°, 43.5°, respectively; the Carr’s index decreased from 43.97 to 37.97, 36.93, respectively; the Hausner ratio decreased from 1.78 to 1.61, 1.59, 1.56, respectively; (ii) the hygroscopicity was decreased from 4.98% to 4.79%, 4.49%, 3.94%, respectively; (iii) the hardness of powder increased from 5718.91 g to 10086.81 g, 12181.30 g, 14613.73 g, respectively; and (iv) the tensile strength increased from 1.8 MPa to 2.6 MPa, 3.65 MPa, 3.75 MPa, respectively. The main mechanisms responsible for the above improvements could be as follows: (i) the MCC coated on the primary particles helped in reducing the cohesion effects of the core particles and increased both total surface area and mechanical interlocking between particles; and (ii) high moisture hinders the free flow of the powders. Therefore, the waterproofing effect of MCC could prevent the water entry into the particles; and (iii) the MCC could increase the surface energy of composite particles, resulting in the higher bonding strength.

MCC is also a commonly used surface modifier. It improves powder properties by employing physical modification techniques, which not only provide a possible solution for drugs that cannot be compressed into integrity tablets but also increase the loading of drugs into tablets while enhancing their disintegration. Consequently, these functionalities are extremely useful for both applications and reducing the use of excipients in tablets. Traditional tablets (that are not modified by surface modifiers) often need a higher percentage of binder, filler, lubricant, and disintegrant to improve the direct compaction properties, resulting in low drug loading. On the other hand, composite powders exhibit improved hygroscopicity, glass transition temperature, and tableting behavior, which are beneficial to form qualified tablets and ensure their stability during storage and transportation.

### 5.2. Binary Modifiers

Lactose, a fragile material, was modified by a binary modifier, i.e., MCC and cornstarch [160]. The proportion of lactose to MCC and cornstarch was 7:2:1, respectively, and they were co-processed by spray drying. The co-processed products exhibited better flow properties and compressibility than that of the pure lactose unprocessed with MCC and cornstarch. Compared with pure lactose, the Hausner ratio of co-processed products decreased from 1.34 to 1.16, and the tensile strength of co-processed products increased from 0.6 MPa to 1.2 MPa. This may be ascribed to the larger inter-particle contact area, the increasing number of bonds in composite particles and the spherical particle shape.

## 6. Mannitol

Mannitol, a versatile excipient with low hygroscopicity and inertness to APIs, is widely employed in tablets and other dosage forms. It has a sweet and cool taste that can cover the unpleasant taste of drugs [161,162]. Therefore, mannitol was developed into many types of granules and mixture granules. It is marketed as a pharmaceutical excipient for the direct tableting of oral disintegration tablets and as a bulking agent and protectant for freeze-drying [163,164]. It also can be used as a modifier in combination with other excipients to produce a multi-function composite excipient by co-processing methods [165,166].

### 6.1. Co-Spray Drying

In the research reported by Al-Khattawi A et al., The aqueous combined mannitol (10%, ***w***/***w***) with NH_4_HCO_3_ (5%, ***w***/***w***) was co-processed by co-spray drying [165]. The ammonium bicarbonate was the pore former, which could be completely removed during spray drying [167]. Compared to the pure mannitol tablets, the co-processed mannitol tablets showed improved porosity, hardness, and disintegration time, and it can be explained as follows: (i) the porosity increased from 0.20% to 0.53%; (ii) the disintegration time decreased from 135 s to 31.67 s; (iii) the hardness increased from 104.17 N to 152.70 N. The reasons for improvement of tablet properties could be attributed to the co-processed mannitol having a higher porosity and plasticity and lower particle density.

### 6.2. Adsorption

Adsorption is a common method for improving the dissolution properties of insoluble drugs by using a carrier with sufficient surface area and pore spaces for particle dispersion and deposition [168,169,170]. In the study conducted by Saffari M et al., sucrose was selected as the templating agent, which was co-spray dried with mannitol to prepare composite products. The concentrations of the two materials in solution were 10% (*w*/*w*) for mannitol and 2% (*w*/*w*) for sucrose. The composite products were washed with ethanol to remove sucrose and produce a porous mannitol carrier. As a result of the application of the carrier, nifedipine and indomethacin showed improved drug loading and dissolution. The results were as follows: (i) the drug loadings improved from 3.2% (*w*/*w*) to 9.1% (*w*/*w*) and 4.1% (*w*/*w*) to 12.6% (*w*/*w*); and (ii) the cumulative drug release improved from 58% to 99% and 50% to 90%. The mechanism of improved dissolution and drug loading could be summarized as follows: (i) the disordered reorganization and dispersion of drug crystals leads to a reduction in particle size of the drug loaded inside the porous mannitol to the nanometer scale; (ii) corresponding improvement in wettability and dispersibility of co-treated particles; (iii) the larger pore volume of porous mannitol is conducive to the entry and penetration of the dissolution medium and improves the contact area and contact rate of the medium, thus, improving the dissolution of the drug. The applications of MCC were summarized in Table 4 [64,133,154,165,166,171,172].

## 7. Others

### 7.1. Polyvinylpolypyrrolidone

Polyvinylpolypyrrolidone (PVPP) is a resin with a highly cross-linked form of the water-soluble polyvinylpyrrolidone polymer. PVPP is insoluble in water and is used in many fields, e.g., DNA extraction, beverage clarification, super-disintegrant, and so on [173,174]. In pharmaceutical formulation, PVPP is used as a super-disintegrant for orally disintegrating tablets due to its capability to absorb water, resulting in rapid swelling. PVPP can also be combined with other surface modifiers, e.g., HPMC, PVP, and HPC, to form binary modifiers, which could significantly improve the tablet disintegration without sacrificing their tabletability [175].

In order to improve the compactibility of MH, Al-Zoubi et al. also employed PVPP VA 64, sodium alginate, and sodium carboxymethylcellulose as surface modifiers and prepared core-shell CPs with varying ratios (MH/surface modifiers, *w*/*w*, 95~97.5/5~2.5) by co-spray drying [28]. The results demonstrated that all three surface modifiers could act as crystal growth inhibitors and significantly improve the compactibility of MH. Compared to MH, which could not be compacted into an intact tablet at pressures between 74 and 444 MPa, all CPs produced strong tablets with high tensile strength (2.07~5.17 MPa) at porosity of 0.15 and showed enhanced tableting parameters, such as 64.9~79.1% higher work of compaction, 7.8~14.5% lower yield pressure, and 10.6~45.5% lower elastic recovery.

In an earlier paper published by Wang ST and coworkers [95], PVPP, lactose 90 M, and HPMC were co-spray dried to improve the tableting properties of lactose. The ratio of the three materials was 89.5:7:3.5, respectively. Compared to the unmodified lactose, the co-processed ternary composite showed improved tableting properties: (i) the AR decreased from 48.7° to 45.0°; (ii) the Hausner ratio decreased from 1.67 to 1.57; (iii) the compaction ratio was decreased from 22.81% to 20.00%; and (iv) the yield pressure was decreased from 179.7 MPa to 113.0 MPa. The mechanism of modification is summarized in Section 2.1.1 and Section 2.2.

### 7.2. Ammonium Bicarbonate

Ammonium bicarbonate (NH_4_HCO_3_) is a commonly used porogen that can be gasified and converted into H_2_O, NH_3_, and CO_2_ when the temperature is above 50 °C [98]. In recent years, it was widely used as the porogenic agent to prepare porous particles by co-spray drying and fluid-bed coating, which provided high hot airflow to completely eliminate the NH_4_HCO_3_. The application of NH_4_HCO_3_ in producing porous particles could simultaneously solve the tableting and dissolution problems of APIs [165,176].

In the report written by Zhou MM et al., the ethanol extract of *Pueraria lobatae Radix* was chosen as the model drug, which was co-spray dried with different amounts of NH4HCO3 to prepare *Pueraria lobatae Radix* porous particles to improve its tabletability and bioavailability. The amounts of NH_4_HCO_3_ were 6.67%, 10.00%, and 13.33%, respectively [98]. Compared to the unprocessed *Pueraria lobatae Radix* powder, the co-processed products exhibited improved properties: (i) the AR decreased from 49.3° to 44.7°, 44.2°, 43.2°, respectively; (ii) the tensile strength was increased from 0.75 MPa to 2.9 MPa, 3.5 MPa, 4.5 MPa, respectively; and (iii) the corresponding tablets dissolution rates improved to twice that of the raw tablets. The improvement reasons could be summarized as follows: first, the morphology of co-processed products exhibited loose, hollow, porous, and spheroidal structures; second, the spheroidal and porous/hollow structures were easily broken under pressure, which could increase the interparticle and intraparticle contact area and result in the formation of a tight combination during compaction; and third, the porous particle compacted to tablets could have higher tablet porosity, leading to the improvement in dissolution by facilitating water infiltration into tablets.

### 7.3. Sodium Lauryl Sulphate

Sodium lauryl sulphate (SLS) is a commonly used surfactant that has the advantage of controlling inter-particle cohesion force and is widely used to increase the wettability and solubility of drugs [177]. In the report published by Solomon S et al., lignin was co-spray dried with SLS in order to improve the compaction properties [178]. The ratio of lignin to SLS was 90:10. Compared to the primary lignin, the compressibility of co-processed products was increased from 25% to 40%. Moreover, when the porosity was 10% and 15%, the tensile strength increased from 1.8 MPa to 2.2 MPa and 1.0 MPa to 1.7 MPa, respectively. The improvement of compressibility could be due to the fact that SLS bonded on the surface of lignin particles, thus, promoting plastic deformation and counteracting elastic deformation.

### 7.4. Magnesium Stearate

Magnesium stearate (MgSt) is the most widely used pharmaceutical lubricant and is known for its friction and sticking reducing effects and cost efficiency [179,180]. MgSt is also popular in particle engineering to improve the flow properties of drug powders. A smaller amount of MgSt as the guest material can significantly enhance the flowability of the host drug and does not have a significant effect on tablet disintegration by the dry coating method [36].

The dry coating method is extensively reported as a promising approach for improving the flow, dispersion, and fluidization of the selected cohesive drug powders [51,151,181,182]. It improves the flow properties of the powders mainly by coating the guest particles on the surface of the host particles in order to reduce powder cohesion.

In the research reported by Qu L et al., a fine ibuprofen powder with remarkable cohesive properties and a low melting point, which can cause sticking and picking during tableting, was chosen as the model drug [132]. MgSt was selected as the surface modifier. The proportion of ibuprofen powder to modifier was 99.9:0.1, 99:1, and 95:5 (*w*/*w*), respectively. They were co-processed by the dry coating method. Compared to the raw ibuprofen powder, the modified products showed improved flow properties: (i) the cohesion decreased from 1.24 kPa to 0.94 kPa, 0.48 kPa, and 0.41 kPa, respectively; and (ii) the flow function (ffc) value increased from 4.0 to 5.0, 8.8, and 10.6, respectively. The ffc is an indicator of powder flowability (ffc < 1, not flowing; 1 < ffc < 2, very cohesive; 2 < ffc < 4, cohesive; 4 < ffc < 10, easy flowing; ffc > 10, free-flowing). (iii) The Carr’s index decreased from 0.37 to 0.27, 0.16, and 0.21, respectively. Additionally, both of the corresponding tablets of raw powder and co-processed powders were dissolved in 5 min. The above phenomena can be analyzed from the following aspects: (i) an extensive MgSt coating layer was formed on the host particle surface, which facilitated enhanced packing of the powder, resulting in the reduction in interparticle cohesion; and (ii) the co-processed particles exhibited relatively smooth surfaces, which were beneficial to enhance the flow properties of modified products.

In another published research, a cohesive lactose monohydrate powder was chosen as the model material, and 1% (*w*/*w*) MgSt was selected as the modifier [183]. They were co-processed by the dry coating technique. Compared to the untreated lactose powders, the dry coated products showed enhanced flow properties. The AR decreased from 64.6° to 38.4°, and the Carr’s index and Hausner ratio decreased from 0.50 and 1.99 to 0.29 and 1.40, respectively. The cohesion decreased from 1.88 kPa to 0.47 kPa, and the flow function (ffc) value was increased from 2.68 to 10.7. The main mechanism responsible for the above phenomena can be as follows: (i) the decrease in strength of interparticle forces after dry coating with MgSt; and (ii) the formation of delaminated MgSt thin films on the surface of host particles could reduce cohesion and friction forces among particles or between particles and walls.

### 7.5. Hydroxypropyl Cellulose

Hydroxypropyl cellulose (HPC), is the product obtained after cellulose hydroxypropylation and is widely used in food and pharmaceutical fields. It is popular in the medicine field for its multifunctionality, e.g., stabilizer, thickener, disintegrant, binder, sustained release material, and so on [184,185,186,187]. Different molecular weights of hydroxypropyl cellulose exhibit different functional properties. The low molecular weight of hydroxypropyl cellulose grades showed better compactibility for its greater plastic deformation and lower post compaction ejection. The high molecular weight of hydroxypropyl cellulose is widely used in sustained and extended formulations because of the ability of retarding the release of the drugs [188].

In a study reported by Shi LM et al., micronized acetaminophen (ACM, Form I) was chosen as the model active pharmaceutical ingredient with poor direct compaction properties, 1–10% *w*/*w* HPC HXF was utilized as the unitary modifier, and they were co-processed by co-spray drying [116]. The co-processed products showed improved tabletability. At 200 MPa, pure acetaminophen and physical mixtures containing up to 40% HPC could not be compacted into intact tablets. In contrast, the composite particles could form strong tablets (tensile strength: 1.9–7.0 MPa) at 200 MPa. Under a certain compaction pressure, the tensile strength values of tablets produced with composite particles containing 1%, 2.5%, 5%, and 10% HPC were 1.51, 1.95, 2.84, 3.57 MPa. The improvement of tabletability could be attributed to the high bonding capability and the good plastic deformation nature of the modifier.

In another reported by Lin X et al., 5–15% HPC EF was dissolved in lactose (pharmatose 80 M) suspension to produce composite core-shell particles by co-spray drying [189]. When compared to the pure particles, the composite particles exhibited improved tabletability: the crushing force was increased from <2.0 N to 27.8~36.4 N; and the tensile strength was increased by 6.7~6.8 times (compaction force: ~60 MPa) and 19.0~22.3 times (compaction force: ~155 MPa). The modification mechanisms were discussed in the previous paragraph. In addition, HPC as a crystallization inhibitor can effectively inhibit the recrystallization of amorphous lactose during direct compaction and storage.

Spherical crystallization is a particle design technique by which crystallization is conducted in one step. It is widely utilized for improving the flowability and compactibility of crystalline drugs [190]. For example, Nokchodchi A et al. reported that 2.1% (*w*/*w*) HPC was dissolved in an acetone–water solution to serve as the crystallization system for improving the flowability and compactibility of naproxen [191]. Compared to the untreated naproxen, the products show improved flow and packing properties: the Carr’s index was decreased from 28.5% to 7.6%; the AR was decreased from 58.8° to 36.4°; and the parameter 1/b of the Kawakita equation and parameter k of Kuno’s equation were increased from 12.5 to 52.6 and 0.0035 to 0.005, respectively, as indicators of tabletability. The larger these values are, the greater the tabletability of the material. The improvements in flowability and tabletability of co-processed particles obtained by spherical crystallization technique were mainly due to the agglomerate’s structures (e.g., shape, size, surface area, and porosity) due to the influence of HPC, which as a crystal modifier, can effectively transform crystalline naproxen to amorphous. The results were in accordance with other studies [192,193]. The applications of other modifiers were summarized in Table 5 [36,98,139,141,165,172,178,194,195,196,197,198,199,200,201,202,203].

## 8. General Modification Mechanisms

### 8.1. The Functional Properties Improved through Modifying the Structure

#### 8.1.1. Particle Structure

**Figure 2:**
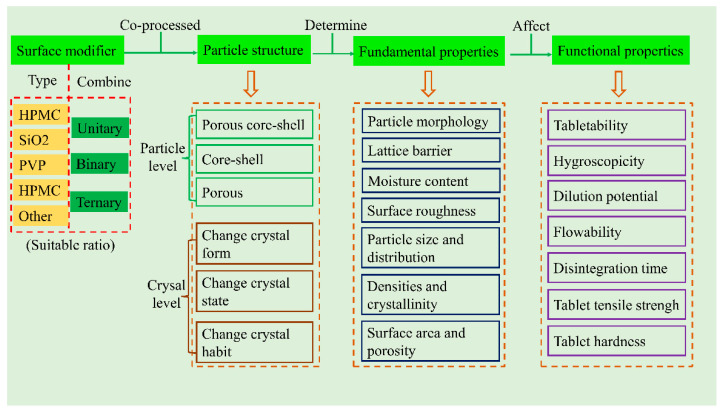
Effects of modifiers on fundamental and functional properties of particles.

Particle properties can be divided into two types: fundamental and functional properties. The fundamental properties include such as particle morphology, size, surface area, porosity, shape, density, and moisture content. The functional properties of the particles include flowability, lubricant sensitivity, tabletability, disintegration, and hygroscopicity, and they are interdependent and determined by fundamental properties [9,22]. In addition, these functional properties are also affected by particle texture properties (such as hardness, springiness, cohesiveness, and resilience). Since fundamental properties of particles are primarily decided by particle structure, it is reasonable to believe that particle structure affects functional properties of particles significantly (Figure 2). Therefore, by designing a special structure for a particle, one can change the particle’s functional properties. In recent years, researchers have utilized co-processing technologies and surface modifiers to obtain composite particle with porous, core-shell, and porous core-shell structures. These special structures were successfully used in direct compaction for improving the key direct compaction properties of active pharmaceutical ingredients.

(i) Core-shell structure, which is one of the most common and important structures for direct compaction can be seen as a structure with internal (core) and external (shell) layers or host (core) and guest (shell) [6,9,18,124]. Core-shell particles are often prepared by dry coating (co-milling) and wet coating (fluid-bed coating, co-spray drying, co-freeze drying, and liquid dispersion) [96,142,204]. Silica (hydrophilic and hydrophobic), microcrystalline cellulose, and magnesium stearate are often utilized as the shell materials to prepare core-shell composite particles through dry coating. Plastic polymers (HPMC, PVP, HPC), mannitol, and lactose are often utilized as the shell materials to prepare core-shell composite particles through wet coating. Core-shell particles with suitable surface modifiers often exhibit a better flowability owing to the fact that surface coating with well-dispersing modifier particles or molecules increases the distance and decreases the contact area of separately coated cohesive host powders, and thus, effectively reduces the van der Waals forces between composite particles. Size enlargement, spheroidization, and surface smoothing of composite particles are also important contributors to the improvement of flowability. The surface layers help to prevent water from penetrating into the core particles, thereby improving the flowability of composite particles. Core-shell particles coated with a layer of highly bonding polymer show improved tabletability, particularly for extremely poorly compactible powders. When coated with a layer of nano-sized silica, the composite particles can provide a larger bonding area and more particle–particle contact points than initial particles without coating. When surface coating forms a certain degree and controls the nature of particle–particle bonding, the mechanical properties of core particles could be ignored. Therefore, the tabletability of raw particles can be effectively modulated and controlled by the coating layer by carefully selecting and combining modifiers (for example, PVPP-HPMC, in this case, PVPP is a super-disintegrant, while HPMC has low hygroscopicity, high glass transition, and high plastic deformation).

(ii) Porous structures have gained a lot of attention for their tremendous potential in improving the dissolution behavior and compactibility of tablet formulations. In terms of their high porosity and high specific surface area, particles with porous structure are widely used in the pharmaceutical field [98,113,165,169]. These particles can be prepared by co-processing of the material with a porogen (a modifier) such as ammonium bicarbonate, camphor, menthol, and thymol [205,206]. The preparation of porous particles through co-processing includes the vacuum drying method and spray drying method. Porous particles show better direct compaction properties because of the increase in porosity and surface roughness, which significantly affects their micromeritic properties, tableting performances, and dissolution [207,208]. As a result, could effectively improve the bonding force and bonding area between the particle surfaces under pressure and, thus, result in strong compacts. High porosity and surface area could provide strong adhesion sites for particles, resulting in less segregation during powder mixtures [209,210]. Porous particles show a better dissolution rate due to water penetration, which facilitates disintegration and dissolution, as well as increased contact area between the drug and the dissolution medium [211,212,213].

(iii) Porous core-shell structures are novel structures containing core-shell and porous structures. Particles with porous core-shell structures can be prepared by co-processing with suitable coating modifiers and porogens. The major co-processing methods are co-spray drying and fluid-bed coating. It has the advantages of both core-shell and porous structures. These benefits and modification mechanisms were discussed in detail above in (i) and (ii).

#### 8.1.2. Crystal Forms and Habits

Crystallization is one of the strategies for particle formation and provides a method by which pharmaceutical materials can be controlled and optimized for specific processing applications, e.g., direct compaction [9,214]. It is not a purification method but, more importantly, an approach to control the polymorphs, crystal habits, and particle size of drugs. A large number of drugs are crystalline in nature and have different crystal habits. Different habits of crystals have different influences on compaction and tableting behavior. The crystal habit of a material can play an influential part in affecting flowability, compactibility, packing, and dissolution.

(i) Crystal forms: Crystalline forms often display irregular shapes (e.g., long needles, thin plate shape, prism shape), which are not good for the flow properties of particles [215]. Crystallization techniques can increase particle size, change particle shape, and reduce inter-particulate friction, thus, leading to the improvement of flowability.

(ii) Crystal habits: Crystal growth and the relative orientation of crystallites are important crystal habits, which are the key factors in deciding the size and shape of crystals and increasing the number of interparticle contact points. The increase in inter-particle contact points could improve the degree of particle deformation, densification, and magnitude and the extent of bond formation during compaction. A small number of effective excipients/modifiers in the crystallization medium can dramatically change the crystal size and shape by blocking the surface of a growing crystal, thus, resulting in preventing its growth and production. Therefore, crystal growth modifiers are often used to modify the structure of particles at particle and crystal levels.

(iii) Crystal states: The crystal states can be divided into two types, such as crystalline and amorphous states. Compared to the crystal state (or the crystal material), the amorphous state (or amorphous material) generally shows improved tabletability due to the tendency for plastic deformation [216]. The addition of modifiers with plastic deformation capability in the crystallization medium or using techniques such as spray drying can effectively improve the proportion of the amorphous form in the final product or generate totally amorphous solids [217].

The amorphous phase usually displays disordered structure and exhibits higher free energy (thermodynamic driving force), thus, resulting in higher apparent water solubility, dissolution rate, and oral absorption. In contrast, the crystalline phase shows poor dissolution behaviors due to its lattice energy barrier. Some modifiers, for example, PVP, a water-soluble polymer, could effectively increase the wettability of active pharmaceutical ingredient particles in addition to inhibiting crystal formation and growth, thus, improving the dissolution of the target drugs.

Surface modifiers (PVP, HPMC, HPC, etc.) were widely utilized as crystal growth inhibitors and effective additives for improving the compression properties of target drugs. The improvement in tabletability of composite particles obtained by co-processing techniques (e.g., co-spray drying, the spherical crystallization technique, co-crystallization techniques, and crystallo-co-agglomeration technique) is mainly due to the agglomerate structure (e.g., shape, size, surface area, and porosity) and the presence of crystal habit, state, and form (amorphous state, spherical form, and size enlargement).

### 8.2. The Synergistic Effect of Co-Processing Methods

**Figure 3:**
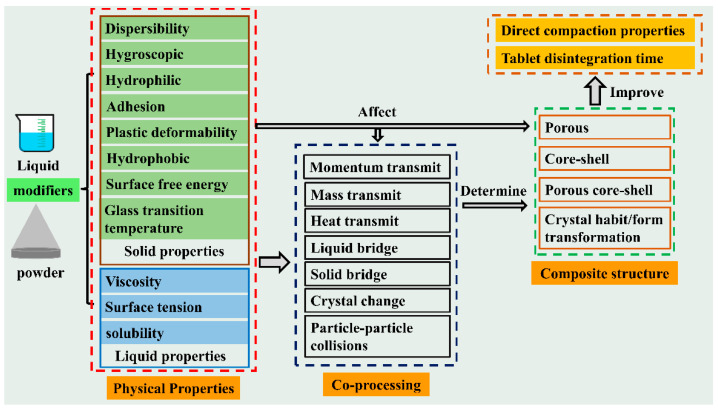
Mechanism of modifiers in improving direct compaction properties.

A surface modifier co-processed with the drug or excipient can effectively improve the physical properties of the drug or prepare high functionality excipients with excellent properties and better application (Figure 3). Co-processing methods, serving as a platform for modifying drug properties, play an important role in successfully enhancing the properties of a drug or an excipient. Fluid-bed coating provides some advantages to modifiers, one of which is the ability to distribute the modifier effectively on the surface of the primary particles. Additionally, the co-processed products exhibit irregular surfaces and large agglomerates. This leads to mechanical interlocking among particles and increasing the contact area during compaction, resulting in enhanced compaction and flow properties and lower lubricant sensitivity. Fluid-bed coatings often have certain requirements for coating liquids, such as their viscosity and surface tension. Thus, they are not suitable for modifiers with low viscosity. Additionally, they are also not suitable for drug powders with high adhesion as they are prone to collapsing during fluidized bed coating. Co-spray drying involves the formulation of an anhydrous powder from slurry by prompt evaporation with hot gases, which can prepare the powder with a specific particle size and moisture content. In the process of spray drying, the crystalline materials (include monobloc drugs and modifier) can be partially or totally transformed into amorphous ones, which is determined by the extent of dissolution and precipitation cycle and the glass forming properties of the materials. Therefore, co-spray drying provides modifiers with the opportunity to enhance tableting properties by using suitable temperature parameters to increase the proportion of amorphous forms with better tableting properties. The products of spray drying usually exhibit spherical structures and smooth surfaces, which are beneficial for producing a synergistic effect in improving flow properties. Owing to the higher inlet temperature and the liquid state of materials, modifiers should have suitable viscosity, surface tension, dispersibility, solubility, and high glass transition temperature. It is not suitable to use modifiers and drugs with low glass transition temperature, which may lead to sticky wall problems. Dry coating is achieved by using co-milling equipment to coat guest particles (modifier particles) on the surface of guest particles (drug or excipient particles), resulting in a significant improvement in their properties. The dry coating method is capable of effectively coating modifiers on the surface of guest particles by generating continuous rotation and grinding, which can enhance the adhesion effect of the modifier. It is frequently used to prepare composite particles with smaller particle size and higher surface free energy. However, it is not suitable for drugs and modifiers that are temperature sensitive due to the heat generated during milling. The freeze-drying process produces a synergistic effect by forming loose aggregates and porous structures during the drying phase. Freeze drying requires modifiers and drugs with suitable freezing points and solid contents.

### 8.3. Others

According to the excipient classification system, surface modifiers can be divided into sugars (mannitol, lactose, xylitol, etc.), salts (calcium carbonate, ammonium bicarbonate, sodium bicarbonate, etc.), silica (nano-silica, colloidal silicon carbonate, hydrophobic or hydrophilic silica, etc.), polymers (microcrystalline cellulose, hydroxypropyl methylcellulose, polyvinylpyrrolidone, etc.) and others. Sugars, for example, lactose and mannitol, are frequently used as template agents to produce porous carriers, which can significantly improve the drug loading and dissolution of target drugs. Silica is a fine and easily dispersed powder that can notably enhance the flowability of the substances by forming a homogenous coating. Polymers, such as HPMC, PVP, HPC, and their solutions have remarkable viscosity; thus, they are widely utilized in the preparation of core-shell composite structure particles by co-spray drying and fluid-bed coating.

The molecular weight of the surface modifier can affect the applications of modifiers. For example, HPMC E3 may be used as a coating material for preparing composite particles with improved DC properties. Additionally, HPMC F4M can serve as a cushioning agent in multi-unit pellet system tableting. HPMC K100M is often selected as a controlled release agent to prolong the release of the drug. Generally, polymers with lower molecular weights exhibit better plasticity, smaller viscosity, and lower glass transition temperatures.

When polymers are used as crystalline inhibitors, the effect tends to become more pronounced as the dosage increases. Furthermore, hydrophilic PVP shows a greater impact on drug crystallinity than HPMC and copovidone [28]. PVP can be linked with the drug via strong hydrogen bonds with the carbonyl (NC_O) group and HPMC with weaker hydrogen bonds through the hydroxyl (−OH) group. The earlier melting and the greater decrease in crystallinity observed at the higher polymer content can be attributed to the increased extent of drug–polymer interactions and increased viscosity, impairing the diffusion of drug molecules into the growing crystals.

The particle size of the surface modifier may affect the flow properties, compaction, and disintegration of target substance. For example, nano-silica, a nanoscale powder, can effectively disperse on the surface of another material forming an agglomerate by dry coating that can remarkably improve the flow properties. Different particle sizes of MCC also exhibit different modification effects, such as, the micronized MCC displays excellent flowability and tabletability.

The thermodynamic properties of the surface modifier play an important role in improving the functionalities of co-processed products. Generally, polymeric modifiers exist in an amorphous semi-crystalline state. The temperature at which the polymer changes from the glassy state to rubbery state is called glass transition temperature (Tg), which is the major thermodynamic property of polymeric modifiers. When the temperature is below the Tg, the polymer modifiers present greater resistance to deformation, and when the temperature is above the Tg, they exhibit decreased resistance to deformation. Therefore, the degree of elastic deformation is mainly determined by their Tg. The higher level of elastic deformation indicates less permanent inter-particulate bonding and shows poor compactibility. Thus, the use of an appropriate polymeric surface modifier can effectively improve the tabletability of co-processed products by co-processing technology.

Some polymers absorb water and cause swelling in particles, which results in breaking. Some polymer modifiers (e.g., L-HPC, PVPP, polyvinyl alcohol, etc.) exert the swelling effect when dissolved in the water and can significantly improve the disintegration time of the drug.

## 9. Future Perspectives and Concluding Remarks

Surface modifiers offer significant advantages and can be used to produce a wider range of APIs/excipients with excellent properties for direct compaction (Table 6). As a result, flowability, tabletability, wettability, solubility, and other properties can be improved. A combination of co-processing technology and the modifier could be used to successfully prepare functional composite particles, such as porous particles, core-shell particles, and porous core-shell particles. However, the selection and combination of modifiers present a certain degree of uncertainty. Therefore, it is necessary to develop a scientific and regulated selection and matching mechanism. Furthermore, the study of the mechanism is insufficiently detailed and fails to offer a comprehensive analysis of the principle and law of the modifier from an analytical perspective. In addition, it is important to explain and explore the mechanism of modification from a quantitative perspective. Moreover, some modifiers and their multiple combinations are not officially recognized by the Pharmacopoeia, which is a major barrier to their successful entry into the market. A collaborative effort is required among academic institutions, excipient manufacturers, and pharmaceutical companies in order to achieve this goal.

In this review, some frequently used modifiers and their binary and ternary composites were summarized. Additionally, they were analyzed in terms of their applications in direct compaction and modification mechanisms. Moreover, the use of suitable modification technology (based upon recent studies) indicates that modifiers could be used to effectively prepare functional composite particles and improve the direct compaction properties of APIs. In addition, this review also provides some potential guidance for formulation scientists involved in the development of tablet dosage forms. In this article, we have summarized the most commonly used pharmaceutical excipients for tablet formulation. Small amounts of excipients can be employed as modifiers through appropriate co-processing techniques thereby enhancing the DC properties of drug powders. As a result, it will provide a novel alternative to conventional tablet formulations, which contain a high proportion of excipients to enhance DC properties.

## Figures and Tables

**Table 1:** The application of HPMC.

Material	Modifier	Type	Processing	Improved Functional Properties	Ref.
Metformin	HPMC-mannitol	Binary modifier	Freeze-dried	Dissolution: disintegration time, ↓, 41%; Tabletability ^(1)^: TS, ↑, 2.5~5.2-fold	[64]
*Andrographis* *paniculate* extract, *Gardenia* extract	HPMC E3 (7%)-mannitol	Binary modifier	Fluid-bed coating	Flowability ^(1)^:AR, ↓, 26.54%; CI, ↓, 36.52%; Flowability (2): AR, ↓, 26.95%; CI, ↓, 37.95%; Tabletability ^(1)^: TS, ↑, 1.54~4.58-fold Drug loading ^(A and G)^: ↑, 75% and 50%	[30]
*Andrographis* *paniculate* extract, *Gardenia* extract	HPMC E3 (7%)-mannitol	Binary modifier	Spray-dried	Flowability ^(1)^:AR, ↓, 29.91%; CI, ↓, 37.77%; Flowability ^(2)^: AR, ↓, 30.29%; CI, ↓, 40.22%; Tabletability ^(1)^:TS, ↑, 2.28~3.07-fold; Drug loading ^(A and G)^: ↑, 75% and 50%	[30]
*Andrographis* *paniculate* extract, *Gardenia* extract	HPMC E3 (7%)-CC	Binary modifier	Fluid-bed coating	Flowability ^(1)^:AR, ↓, 20.30%; CI, ↓, 40.82%; Flowability ^(2)^: AR, ↓, 21.95%; CI, ↓, 39.22%; Tabletability ^(1)^: TS, ↑, 3.28~5.98-fold Drug loading ^(A and G)^: ↑, 75% and 25%	[30]
*Andrographis* *paniculate* extract, *Gardenia* extract	HPMC E3 (7%)-CC	Binary modifier	Spray-dried	Flowability ^(1)^:AR, ↓, 10.57%; CI, ↓, 21.65%; Flowability ^(2)^: AR, ↓, 12.42%; CI, ↓, 22.64%; Tabletability ^(1)^: TS, ↑, 2.61~5.11-fold Drug loading ^(A and G)^: ↑, 75% and 25%	[30]
Ibuprofen	HPMC	Unitary modifier	Fluid-bed coating	Flowability ^(2)^: flow rate: ↑, 1.08~2.5-fold	[96]
Three kinds of alcohol extracted medicinal powders	HPMC (7%)	Unitary modifier	Fluid-bed coating	Flowability ^(1)^: AR↓, 14.89%; 25.38%; 31.00%; Flowability ^(2)^: AR↓, 14.30%; 25.97%; 16.38%; Tabletability ^(1)^: AUTCC↑, 2.20-fold; 40.60- fold; 0→8.786 MPa.kN; Tabletability ^(2)^: AUTCC↑, 2.50-fold; 30.70- fold; 0→8.786 MPa.kN; Hygroscopicity ^(2)^: f(ZR)↓, 16.94%	[97]
The Andrographis herba extract	HPMC (6%)	Unitary modifier	Fluid-bed coating	Flowability ^(1)^: AR↓, 27.11%; Flowability ^(2)^: AR↓, 26.88% Tabletability ^(1)^: AUTCC↑, 1.96-fold; Tabletability ^(2)^: AUTCC↑, 1.95-fold	[18]
The Andrographis herba extract	HPMC (9%, 12%)	Unitary modifier	Fluid-bed coating	Flowability ^(2)^: AR↓, 28.92%; 30.48%; Tabletability ^(2)^: AUTCC↑, 2.07- fold; 2.26-fold	[18]
*Zingiberis rhizoma* extracted powder	HPMC E3 (7%)	Unitary modifier	Fluid-bed coating	Flowability ^(1)^: AR↓, 25.76%; Flowability ^(2)^: AR↓, 20.26% Tabletability ^(1)^: AUTCC↑, 0→8.786 MPa.kN Tabletability ^(2)^: AUTCC↑, 0→8.786 MPa.kN Hygroscopicity ^(1)^: f↓, 8.72%; Hygroscopicity ^(2)^: f↓, 16.94%	[6]
*Zingiberis rhizoma* extracted powder	HPMC E3 (7%)-SiO_2_ (1%)	Binary modifier	Fluid-bed coating	Flowability ^(1)^: AR, No significant improvement Flowability ^(2)^: AR↓, 23.17; Tabletability ^(1)^: AUTCC↑, 6.07-fold Tabletability ^(2)^: AUTCC↑, 0→14.401 MPa.kN Hygroscopicity ^(1)^: f↓, 15.71%; Hygroscopicity ^(2)^: f↓, 20.77%	[6]
*Zingiberis rhizoma* extracted powder	HPMC E3 (1.4%)-mannitol (5.6%)	Binary modifier	Fluid-bed coating	Flowability ^(1)^: AR↓, 20.52%; Flowability ^(2)^: AR↓, 22.18% Tabletability ^(1)^: AUTCC↑, 0→6.405 MPa.kN Tabletability ^(2)^: AUTCC↑, 0→6.405 MPa.kN Hygroscopicity ^(1)^: f↓, 0.53%; Hygroscopicity (2): f↓, 7.83%	[6]
The ethanol extract of pueraria lobatae radix	NH_4_HCO_3_ (6.67%, 10.00%, 13.33%)	Unitary modifier	Spray-dried	Flowability ^(2)^: AR, No significant improvement Tabletability ^(2)^: TS↑, 2.43-fold; 3.16~3.40-fold; 4.32-7.03-fold Disintegration: dissolution rate↑, ~2-fold	[98]
lactose, corn starch, mannitol,	HPMC	Unitary modifier	Spray-dried	Tabletability ^(1)^: TS↑, 2.43-fold; 4.20~6.28-fold; 1.63~4.12-fold; 1.81~2.69-fold; 1.34~1.63-fold; 1.05~1.24 -fold; Flowability: AR, 43 ± 0.15~48 ± 0.25 Tabletability ^(2)^: TS↑, 2.43-fold	[5]
Starch	HPMC-PVPP (3.5%)	Binary modifier	Spray-dried	Disintegration time ^(C)^: ↓, 4.77–7.58%	[5]
Calcium hydrogen phosphate dihydrate (19%~44%)	HPMC E3 (3.5%~10.5%)	Unitary modifier	Spray-dried	Flowability: AR, 29 ± 0.19~34 ± 0.15°; Tabletability: TS ^(a)^, 2.20 ± 0.04~3.55 ± 0.01 MPa; Disintegration time ^(b)^: 11.33 ± 0.08~59.67 ± 0.07 min	[5]
Calcium hydrogen phosphate dihydrate	HPMC E3 -PVPP (3.5%)	Binary modifier	Spray-dried	Disintegration time ^(C)^: ↓, 0.35–75.82%	[5]
Starch (19%~44%)-	HPMC E3 (3.5%~10.5%)	Binary modifier	Spray-dried	Flowability: AR, 29 ± 0.31~34 ± 0.13°; Tabletability: TS ^(a)^, 3.15 ± 0.04~5.27 ± 0.05 MPa; Disintegration time ^(b)^: 6.06 ± 0.03~13.29 ± 0.01 min	[5]
Metformin	HPMC vlV -lactose-	Binary modifier	Freeze-dried	Disintegration time ^(2)^: ↓, 24.68%; 50.00%	[44]
Lactose	HPMC	Unitary modifier	Dry coating	Drug loading ^(2)^: ↑, 46.15%	[99]
Metformin hydrochloride	HPMC E3 (2.5%, 5%)	Unitary modifier	Spray-dried	Tabletability ^(2)^: TS ↑, 0→1.89, 2.67 MPa; 0→2.65, 5.17 MPa; 0→3.15, 2.81 MPa; 0→1.62, 2.00 KPa; 0→2.07, 2.15 KPa; Tabletability ^(2)^: TS ↑, 192-fold	[28]
Carvedilol Matrix tablets	HPMC (K 4 M)	Unitary modifier	Dry coating	Tabletability ^(2)^: TS↑, 1.32-fold	[100]
Mannitol, lactose, anhydrous dibasic calcium phosphate , calcium carbonate Chitosan	HPMC E3 (7%)	Unitary modifier	Spray-dried	Flowability ^(1)^: AR↓, 36.04%; 28.31%; 28.57%; 29.87%; 30.26% Flowability ^(2)^: AR↓, 11.51%; 23.45%; 28.71%; 26.60%; 7.79% Tabletability ^(1)^: Tableting ratio↓, 0.94%; 15.68%; 11.90%; 26.42%; 2.21%; Tabletability ^(2)^: Tableting ratio↓, 10.62%; 5.74%; 23.67%; 33.05%; 15.70%	[62]

Unlabeled data indicate that they were compared with the physical mixture; ^(1)^ compared with the unprocessed particles; ^(2)^ compared with the processed particles without additive; ^(3)^ and compared with the particles without co-processing. ^(a)^ These values were determined under the compaction force of 176 MPa; ^(b)^ Tablets having a breaking force of 60 N (a commonly acceptable value for commercially available tablets) were used for the determination; ^(c)^ compared with the processed particles without PVPP. Tableting ratio: describes the deformation behavior of a material in the tableting process; the smaller the value, the better the compressibility and compactibility of the material; CC: calcium carbonate; CI: Carr’s index; and f: equilibrium hygroscopic moisture content. ^A^: *Andrographis paniculate extract*; ^G^: *gardenia extract*; P, ethyl alcohol extracted powder of *Poria*; PLR, ethyl alcohol extracted powder of *Puerariae Lobatae Radix*; ZR, ethyl alcohol extracted powder of *Zingiberis rhizoma*.

**Table 2:** The application of PVP.

Material	Modifier	Type	Processing	Improved Functional Properties	Ref.
Acetaminophen	PVP K30 (1.25%, 1.5 %, 5%)	Unitary modifier	Spray-dried	Tabletability ^(2)^: The same compaction (kN), crushing strength↑, ~3.90-fold; ~5.00-fold; ~7.00-fold; Dissolution ^(2)^: Disintegration time↓, 36.61%; 52.29%; 74.31%; Dissolution ^(3)^: Disintegration time↓, 65.48%; 73.60%; 85.79%	[108]
Silicon dioxide	PVP	Unitary modifier	Dry coating	Tabletability ^(1)^: TS, ↑, 0→2.25 kN	[116]
Paracetamol	PVP (5%)- Lactose (20%)	Binary modifier	Spray-dried	Tabletability ^(2)^: TS, ↑, 1.67-fold; Tabletability ^(3)^: TS, ↑, 1.73-fold Flowability ^(3)^: FFC↑, 2.23-fold^;^ Compared with adding PVP and lactose alone, adding PVP and lactose at the same time FFC↑, 1.12-fold; TS↑, 3.18-fold	[115]
Lactose	PVP K30(1 %, 2%, 3%)	Binary modifier	Spray-dried	Flowability ^(2)^: AR↓, 6.84%; 10.60%; 11.70%; CI↓, 22.68%; 30.00%; 21,22%; Flowability ^(3)^: AR↓, 25.57%; 28.57%; 29.54%; CI↓, 32.55%; 38.94%; 31.28%;	[113]
Curcumin	PVP K30(1 %, 2%, 3%) -lactose	Binary modifier	Spray-dried	Dissolution ^(2)^: Cumulative dissolution percentage (%) ↑, at 90 min, 3.31-fold, 3.77-fold, 3.58-fold; Dissolution ^(3)^: Cumulative dissolution percentage (%) ↑, at 90 min, 3.74-fold, 4.26-fold, 4.04-fold	[113]
Leflunomide	PVP	Unitary modifier	Freeze-drying	Dissolution: ↑, Dissolution efficiency (%), At 10 min, 60 min, 120 min, 0→10.73; 0→61.46; 1.05→78.44	[114]
Paracetamol	PVP (5%)- Mannitol (20%)	Binary modifier	Spray-dried	Tabletability ^(1)^: TS, ↑, 8.00-fold; 16.00-fold; 10.17-fold	[117]
Three kinds of water extracted medicinal powders (A, G, GF)	PVP	Unitary modifier	Fluid-bed coating	Flowability ^(1)^: AR↓, 32.47%; 24.81%; 33.77% Flowability ^(2)^: AR↓, 32.51%; 24.30%; 32.72% Tabletability ^(1)^: AUTCC↑, 1.31-fold; 1.64-fold; 3.07-fold Tabletability ^(2)^: AUTCC↑, 1.36-fold; 1.99-fold; 3.12-fold	[97]
Ibuprofen	PVP (10%) MgSt (0.1–5%)	Binary modifier	Dry coating	Tabletability ^(2)^: TS↑, 1.23-fold; Flowability ^(2)^: CI↓, 29%	[36]
Metformin hydrochloride	PVP K30 (5.0%)	Unitary modifier	Spray-dried	Tabletability ^(2)^: TS ↑, 0→1.62 KPa; work of compaction, ↑, 72.8%; elastic recovery, ↓, 1.5%; tablet tensile strength at porosity 0.15, ↑, from 0 to 2.00 MPa.	[28]
Desloratadine	PVP	Unitary modifier	Spray-dried	Dissolution ^(3)^: Apparent solubility↑, 0→1.62, 2.00 KPa	[118]
Artemisinin	PVP (1:1)	Unitary modifier	Spray-dried	Dissolution: at 30 min, relative dissolution rate ^(1)^ ↑, 3.17-fold; relative dissolution rate ^(2)^ ↑, 1.78-fold, relative dissolution rate ^(3)^ ↑, 0 →4.78; at 30 min, percent dissolution efficiency ^(1)^ (%) ↑, 3.16-fold; percent dissolution efficiency ^(2)^ (%) ↑, 1.72-fold; percent dissolution efficiency ^(3)^ (%) ↑, 4.78-fold at 30 min, concentration of drug dissolved ^(1)^ (μg/mL) ↑, 3.15-fold, concentration of drug dissolved ^(2)^ (%) ↑,1.74-fold, concentration of drug dissolved ^(3)^ (%) ↑, 4.78-fold	[119]
Artemisinin	PVP (1:2, 1:4, 1:6)	Unitary modifier	Spray-dried	Dissolution: at 30 min, relative dissolution rate ^(2)^ ↑, 2.16-fold, 2.43-fold, 2.90-fold; relative dissolution rate ^(3)^ ↑, 0→5.78; 0→6.52; 0→7.76; at 30 min, percent dissolution efficiency ^(2)^ (%) ↑, 2.09-fold, 2.35-fold, 2.80-fold; percent dissolution efficiency ^(3)^ (%) ↑, 5.78-fold, 6.52-fold, 7.76-fold; at 30 min, concentration of drug dissolved ^(2)^ (%) ↑, 2.08-fold, 2.35-fold, 2.80-fold; concentration of drug dissolved ^(3)^ (%) ↑, 5.78-fold, 6.52-fold, 7.77-fold	[119]
Telmisartan	PVP K30 (1:5)	Unitary modifier	Spray-dried	Dissolution: at 5 min, percentage of drug dissolved ^(2)^ ↑, 4.05-fold; percentage of drug dissolved^(3)^ ↑, 16.17-fold; at 15 min, percentage of drug dissolved ^(2)^ ↑, 3.63-fold; percentage of drug dissolved^(3)^ ↑, 19.47-fold; at 5 min, percentage of dissolution efficiency ^(2)^ ↑, 4.15-fold; percentage of dissolution efficiency ^(3)^ ↑, 13.46-fold; at 15 min, percentage of dissolution efficiency ^(2)^ ↑, 3.85-fold; percentage of dissolution efficiency ^(3)^ ↑, 16.98-fold; at 5 min, percentage of drug released ^(2)^ ↑, 3.67-fold; percentage of drug released ^(3)^ ↑, 8.25-fold at 15 min, percentage of drug released ^(2)^ ↑,3.94-fold; percentage of drug released ^(3)^ ↑, 21.00-fold Saturation solubility ^(2)^ (mg = mL) ↑, 11.45-fold Saturation solubility ^(2)^ (mg = mL) ↑, 21.40-fold	[120]
Telmisartan	PVP- Aerosil200/Sylysia350 (1:5:2)	Binary modifier	Spray-dried	Dissolution: at 5 min, percentage of drug dissolved ^(2)^ ↑, 4.89-fold, 5.65-fold; percentage of drug dissolved ^(3)^ ↑, 19.58-fold, 22.57-fold; at 15 min, percentage of drug dissolved ^(2)^ ↑, 4.70-fold, 5.59-fold; percentage of drug dissolved ^(3)^ ↑, 25.26-fold, 30.03-fold; at 5 min, percentage of dissolution efficiency ^(2)^ ↑, 4.58-fold, 5.18-fold; percentage of dissolution efficiency ^(3)^ ↑, 14.86-fold, 16.81-fold; at 15 min, percentage of dissolution efficiency ^(2)^ ↑, 4.79-fold, 5.44-fold; percentage of dissolution efficiency ^(3)^ ↑, 21.12-fold, 23.89-fold; at 5 min, percentage of drug released ^(2)^ ↑, 4.00-fold, 4.57-fold; percentage of drug released ^(3)^ ↑, 14.00-fold, 16.00-fold; at 15 min, percentage of drug released ^(2)^ ↑,4.56-fold, 5.56-fold; percentage of drug released ^(3)^ ↑, 20.5-fold, 25.00-fold; Saturation solubility ^(2)^ (mg/mL) ↑, 17.65-fold, 25.56-fold Saturation solubility ^(2)^ (mg/mL) ↑, 33.00-fold, 47.79-fold	[120]
Loratadine	PVP K30	Unitary modifier	Dry coating	Dissolution: Solubility ^(1)^ ↑, 1.17-fold; Solubility ^(3)^ ↑, 1.19-fold; at 60 min, percentage of drug dissolved ^(1)^ ↑, 1.22-fold, percentage of drug dissolved ^(3)^ ↑, 3.33-fold,	[121]
Irbesartan	PVP-sodium dodecyl sulfate	Binary modifier	Anti-solvent precipitation Spray-dried	Dissolution: at 30 min, percentage of drug dissolved ^(1)^ ↑, 8%→100% percentage of drug dissolved ^(3)^ ↑, 40%→100%	[122]
*Zingiberis rhizoma* extracted powder	PVP K30 (7%)	Unitary modifier	Fluid-bed coating	Flowability ^(1)^: AR↓, 26.88%; Flowability ^(2)^: AR↓, 23.49%; Tabletability ^(1)^: AUTCC↑,0→9.279 MPa.kN; Tabletability ^(2)^: AUTCC↑,0→9.279 MPa.kN; Hygroscopicity ^(1)^: f↓, 9.82%; Hygroscopicity ^(2)^: f↓, 14.63%	[6]
Effervescent tablets	PVP K30 (6%)	Unitary modifier	Fluid-bed coating	Flowability ^(1)^: AR↓, 7.42%; Flowability ^(3)^: AR↓, 33.43%; Tabletability ^(1)^: TS↑, 2.09-fold; Tabletability ^(3)^: TS↑, 2.30-fold; Sticking of citric acid (mg) ^(1)^ ↓, 70.83→0.24; Sticking of citric acid (mg) ^(3)^ ↓, 22.80→0.24	[123]

Unlabeled data indicate that they were compared with the physical mixture; ^(1)^ compared with the physical mixture; ^(2)^ compared with the processed particles without additive; ^(3)^ compared with the unprocessed particles; A, water extracted powder of *Andrographis herba*; G, water extracted powder of *Ganoderma*; GF, water extracted powder of *Gardeniae fructus*.

**Table 3:** The application of SiO_2_.

Material	Modifier	Type	Processing	Improved Functional Properties	Ref.
*Zingiberis rhizoma* extracted powder	SiO_2_ (1%)	Unitary modifier	Freeze-dried	Flowability ^(3)^: ffc, ↑, mean↑, 2.60-fold; Mean ffc: 4.00→10.06 Tabletability ^(3)^: TS↓, 28.57%	[142]
Erythritol	Porous SiO_2_ (2:1)	Unitary modifier	Dry coating	Dissolution: Disintegration time↓, 40%; Tabletability ^(1)^: TS↑, 28.57%	[143]
*Zingiberis rhizoma* extracted powder	SiO_2_ (1:0.06, 1:0.5, 1:0.25)	Unitary modifier	Liquid dispersion	Flowability ^(1)^: AR↓, 5.30%, 18.54%; Flowability ^(3)^: AR↓, 7.94%, 20.82%, 18.24%; Tabletability ^(1)^: CR↑, 1.25%; Tabletability ^(3)^: CR↓, 7.69%, ↑, 1.92%, ↑, 3.85%	[133]
Ibuprofen 50	SiO_2_	Unitary modifier	Dry coating	Dissolution: time for 80% of drug to dissolve↓, 12min→3min; Flowability ^(3)^: AR↓, 19.15%, FFC↑, 2.96-fold; Drug loading ^(3)^: at 60%, AR:38°, TS: 3.40 MPa, FFC = 8 (at 60%, AR:47°, TS:2.30, FFC = 2.7)	[25]
Ibuprofen powder	SiO_2_-PVP 40 (4:1)	Binary modifier	Dry coating	Flowability ^(2)^: AR↓, 28.30%, FFC↑, 6.10-fold, Cohesion (KPa) ↓, 40% Flowability ^(3)^: AR↓, 32.07%, FFC↑, 5.90-fold, Cohesion (KPa) ↓, 42.50%; Dissolution ^(2)^: Dissolution rate↑, 2.00-fold; Dissolution ^(3)^: Dissolution rate↑, 3.00-fold	[53]
Ibuprofen (Ibu50, Ibu90)	SiO_2_ (1%-M5P, Aerosil R972P)	Unitary modifier	Dry coating	Flowability ^(3)^: FFC↑, hydrophilic M5P ↑, 1.38-fold, 2.67-fold hydrophobic Aerosil R972↑, 5.00-fold, 3.11-fold	[140]
Ibuprofen, mannitol, lactose	SiO_2_ (1% -Aerosil R972P, Aerosil A200)	Unitary modifier	Dry coating	Flowability ^(3)^: FFC↑, hydrophobic Aerosil R972 ↑, 3.33-fold, 1.74-fold, 1.71-fold; hydrophilic Aerosil A200↑, \, 2.28-fold, 1.84-fold	[144]
Cornstarch	1%, 0.1%, silica EH-5; 20%, silica COSMO-55	Unitary modifier	Dry coating	Flowability ^(3)^: AR↓, silica EH-5 ↓, 36.54%, 34.62% silica COSMO-55↓, 13.46%	[51]
Danshen, Notoginseng, bornel, formulated	1%-silica nanoparticles	Unitary modifier	Dry coating	Tabletability ^(3)^: Compressibility↓, 56.64%, 24.04%, 7.60%, 63.59% TS↑, 1.67-fold, 1.75-fold, 1.53-fold, 1.74-fold; Flowability ^(3)^: AR↓, 53.45%, 27.27%, 16.67%, 44.33%	[145]
MCC (particle size 20, 25, 30, 35 μm)	Hydrophobic (Aerosil R972P), hydrophilic (Aerosil A200) silicas	Unitary modifier	Dry coating	Flowability ^(3)^: FFC↑, hydrophobic Aerosil R972P ↑, 1.70-fold, 2.00-fold, 2.78-fold, 3.00-fold	[33]
Acetaminophen (micronized and coarse)	SiO_2_ (M5P, R972P)	Binary modifier	Dry coating	Flowability ^(3)^: FFC↑, hydrophobic Aerosil R972P ↑, 2.20-fold, 1.88-fold hydrophilic M5P↑, 3.50-fold, 3.09-fold	[140]
Microcrystalline cellulose	Colloidal silica	Binary modifier	Dry coating	Flowability ^(3)^: Flowability energy↑, 1, 92-fold	[146]
API	Silica colloidal anhydrous-MCC-MgSt	Ternary modifier	Dry coating	Flowability: FFC↑, 2.00~2.50-fold; Drug loading: ↑, 50%→80%	[147]
EC	Colloidal silicon (1%)-lactose (5%)	Binary modifier	Dry coating	Sustained drug release: Dissolution rate↓, 4.00-fold; Flowability ^(3)^: AR↓, 11.70%	[148]
CaCO3	Aerosil nanoparticle	Unitary modifier	Dry coating	Flowability ^(3)^: AR↓, 12.76%, CI↓, 12.24%	[149]
Fenofibrate	Hydrophilic nano-silica (M5P) (0.1%, 0.17%, 1%)	Unitary modifier	Dry coating	Flowability ^(3)^: AR↓, 45.22%, 45.40%, 39.40%; FFC↑, 3.57-fold, 3.57-fold, 2.16-fold	[150]
Potassium chloride	Silica	Unitary modifier	Dry coating	Flowability ^(3)^: Flow function coefficient↑, 1.26-fold; Cohesion↓, 25.53%	[141]
Avicel PH-102	Nano-silica (1%)	Unitary modifier	Dry coating	Flowability ^(3)^: Flow function coefficient↑, 3.00-fold; Tabletability(3): TS↑, 1.26-fold	[14]
MCC	Silica nanoparticles (0.1%, 0.5%, 1%, 2%)	Unitary modifier	Dry coating	Flowability ^(3)^: Flow factor↑, 1.20-fold, 3.20-fold, 4.20-fold, 5.20-fold	[151]
Acetaminophen	Silica nanoparticles (0.1%)- MCC PH102, MCC PH105	Binary modifier	Dry coating	Flowability ^(3)^: Flow function coefficient↑, At drug loading 20%, 40%, 60%, 80%, PH102↑, 3.02-fold, 4.30-fold, 5.00-fold, 5.00-fold; PH105↑, 4.20-fold, 3.65-fold, 3.70-fold, 3.25-fold; Tabletability ^(3)^: TS↑, At drug loading 20%, 40%, 60% PH102↑, 1.24-fold, 1.08-fold, 1.33-fold; PH105↑, 1.61-fold, 1.29-fold, 1.38-fold	[34]
Micronized acetaminophen, coarse acetaminophen, micronized ibuprofen	nano-silica (hydrophobic, hydrophilic)	Unitary modifier	Dry coating	Flowability ^(3)^: Flow function coefficient↑, hydrophobic Aerosil R972P↑, 3.80-fold, 4.41-fold, 3.90-fold; hydrophilic CAB-O-SIL M5P ↑, 2.44-fold, 2.96-fold, 3.15-fold; Tabletability ^(3)^: TS↑, hydrophobic Aerosil R972P↑, 1.50-fold, 1.07-fold, 1.09-fold; hydrophilic CAB-O-SIL M5P ↑, 3.00-fold, 1.43-fold, 1.08-fold	[135]
Micronized acetaminophen, Avicel PH105, Pharmatose 450 M	Silica	Unitary modifier	Dry coating	Flowability ^(3)^: Flow function coefficient↑, 2.50-fold, 2.37-fold, 6.33-fold; Tabletability ^(3)^: compressibility (%) ↓, 40.00%, 54.05%, 68.97% Drug loading at 60% had suitable; flowability and tabletability (FFC > 8, TS = 2)	[35]
Ibuprofen powder	Silica-R972 (1%)	Unitary modifier	Dry coating	Flowability: Cohesion ^(3)^ ↓, 81.95%; flow function ^(3)^ ↑, 5.14-fold	[139]

Unlabeled data indicate that they were compared with the physical mixture; ^(1)^ compared with the unprocessed particles; ^(2)^ compared with the processed particles without additive; and ^(3)^ compared with the particles without co-processing.

**Table 4:** The application of MCC and mannitol.

Material	Modifier	Type	Processing	Improved Functional Properties	Ref.
Crospovidone	MCC- sodium chloride	Unitary modifier	Fluid-bed Freeze dryer	Tabletability ^(1)^: Porosity↑, 2.14-fold, 2.57-fold; TS↑, 1.57-fold, 9.28-fold	[154]
Sacubitril valsartan	MCC	Unitary modifier	Spray-dried	Dissolution ^(1)^: solubility↑, 11.5-fold, 3.12-fold; Bioavailability ^(1)^: Relative bioavailability %↑, 14.49-fold, 11.21-fold, 5.64-fold, 1.98 -fold; Bioavailability ^(2)^: Relative bioavailability %↑, 1.54-fold, 1.11-fold	[171]
*Zingiberis rhizoma* extracted powder	MCC (1:0.06, 1:0.25)	Unitary modifier	Dry coating	Flowability ^(3)^: AR↓, 1.07%, 4.94%; Tabletability ^(3)^: CR↓, 17.41%, 18.15%	[133]
Curcumin	MCC	Unitary modifier	Fluid-bed	Flowability ^(3)^: Carr’s index↓, 84.00%; Hausner ratio↓, 46.12%	[172]
Indomethacin and Nifedipine	Mannitol (porous)	Unitary modifier	Spray-dried	Dissolution: The area under dissolution curve↑; Cumulative drug release ^(1)^ ↑, at 10 min, ↑, 2.79-fold, 2.00-fold; balance drug release ^(1)^ ↑, 2.04-fold, 1.69-fold; Drug loading ^(3)^: ↑, 2.84-fold, 3.07-fold	[166]
Metformin	HPMC-mannitol	Binary modifier	Freeze-dried	Dissolution: DT, ↓,41%; Tabletability ^(3)^: TS, ↑, 2.5~5.2-fold	[64]
Mannitol	NH_4_HCO_3_ (5% *w*/*v*)	Binary modifier	Spray-dried	Dissolution: Porosity ^(3)^ ↑, 2.5~5.2-fold; Disintegration time ^(3)^ ↑, 50%~70%; Tabletability ^(3)^: Tablet hardness, ↑, 1.46-fold	[165]

Unlabeled data indicate that they were compared with the physical mixture; ^(1)^ compared with the unprocessed particles; ^(2)^ compared with the processed particles without additive; and ^(3)^ compared with the particles without co-processing.

**Table 5:** The application of other modifiers.

Material	Modifier	Type	Processing	Improved Functional Properties	Ref.
Paracetamol	Alpha-lactose-hydrate	Unitary modifier	Spray-dried	Tabletability ^(1)^: TS, ↑, 2.9-fold	[194]
Propranolol	lactose-HPMC (1:1)	Binary modifier	Spray-dried	Tabletability ^(1)^: Hardness ↑, 1.22-fold; Flowability ^(1)^: Flow time ↑, 1.33-fold	[195]
Lactose monohydrate	PEG 4000-popoxamer	Binary modifier	Fluid-bed granulation	Flowability ^(3)^: Flow rate ↑, 7.73-fold; compressibility index↑, 4.33-fold; Hausner ratio↑, 1.42-fold; Tabletability ^(1)^: TS ↑, 2.83-fold	[196]
Curcumin	MCC-PVP	Unitary modifier	Fluid-bed coating	Flowability ^(3)^: Carr’s index↓, 84.00%; Hausner ratio↓, 46.12%	[172]
Curcumin	Lactose-PVP	Unitary modifier	Fluid-bed coating	Flowability ^(3)^: Carr’s index↓, 6.67%; Hausner ratio↓, 9.13%	[172]
Lactose	Magnesium stearate	Unitary modifier	Spherical agglomerates	Tabletability ^(3)^: TS ↑, 3.50-fold; Flowability ^(3)^: Flow time↑, 2.00-fold	[197]
Mannitol	NH_4_HCO_3_ (5% *w*/*v*)	Unitary modifier	Spray-dried	Dissolution: Porosity ^(3)^ ↑, 2.5~5.2-fold; Disintegration time ^(3)^ ↑, 50%~70%; Tabletability ^(3)^: Tablet hardness, ↑, 1.46-fold	[165]
*Pueraria lobatae* *radix*	NH_4_HCO_3_ (6.67%, 10%, 13.33%)	Unitary modifier	Spray-dried	Flowability ^(3)^: AR↓, 9.33%, 10.34%, 12.37%; CI↑, 1.28-fold, 1.28-fold, 1.27-fold; HR↑, 1.21-fold, 1.20-fold, 1.19-fold; Flowability ^(2)^: AR↓, 1.11%, 2.21%, 4.42%; CI↑, 1.01-fold, 1.00-fold, 1.00-fold; HR↑, 1.01-fold, 1.01-fold, 1.00-fold; Tabletability ^(3)^: TS↑, 3.42-fold, 4.29-fold, 7.71-fold; Tabletability ^(2)^: TS↑, 1.50-fold, 1.88-fold, 3.38-fold	[98]
*Pueraria lobatae* *radix*	NH_4_HCO_3_ (10%)	Unitary modifier	Spray-dried	Dissolution ^(3)^: Dissolution rate↑, 2.00-fold	[98]
Lignin	Sodium lauryl sulphate (SLS)	Unitary modifier	Spray-dried	Tabletability ^(3)^: TS↑, 1.33-fold	[178]
Chlorzoxazone	HPC-Eudragit S100	Binary modifier	Crystallo-co-agglomeration	Flowability ^(3)^: CI↑, 47.87%; Tabletability ^(3)^: TS↑, 3.33-fold Dissolution ^(3)^: Dissolution rate↑, 2.00-fold	[198]
Potassium chloride	Leucine (0.5%,1%, 2%, 5%, 10%)	Unitary modifier	Dry coating	Flowability ^(3)^: Flow function coefficient↑, 1.37-fold, 2.37-fold, 2.42-fold, 2.84-fold, 1.84-fold; Cohesion↓, 41.70%, 62.50%, 64.58%, 68.75%, 72.92%	[141]
Lactose	Silica	Ternary modifier	Spray-dried	Tabletability ^(3)^: TS↑, 1.89-fold	[199]
Ketoprofen	Croscarmellose, crospovidone, starch glycolate	Binary modifier	Spray-dried	Tabletability ^(3)^: TS↑, 3.33-fold; Dissolution ^(3)^: Ketoprofen effectively in 20 min	[200]
Dibasic calcium phosphate	Anhydrous polyethylene glycol-crospovidone	Binary modifier	Dry coating	Dissolution ^(3)^: Disintegration time↓, 48.13%; Cumulative drug release↑, 18.48%	[201]
Ibuprofen	MgSt (0.1%, 1%, 5%)	Unitary modifier	Dry coating	Flowability: Cohesion ^(1)^ ↓, 7.65%, 52.94%, 59.80%; Cohesion ^(3)^ ↓, 29.22%, 74.51%, 81.37%; flow function ^(1)^ ↑, 1.14-fold, 2.02-fold, 2.43-fold; flow function ^(3)^ ↑, 1.24-fold, 2.19-fold, 2.64-fold	[36]
Ibuprofen powder	MgSt, l-leucine	Unitary modifier	Dry coating	Flowability: Cohesion ^(3)^ ↓, 20.30%, 30.08%, 81.95%; flow function ^(3)^ ↑, 1.14-fold, 1.44-fold, 5.14-fold	[139]
Fine lactose powder	MgSt	Unitary modifier	Dry coating	Flowability ^(3)^: higher dispersive energy	[202]
MCC-E 50M	MgSt (1%)	Unitary modifier	Dry coating	Flowability ^(3)^: CI↓, 41.62%	[203]

Unlabeled data indicate that they were compared with the physical mixture; ^(1)^ compared with the unprocessed particles; ^(2)^ compared with the processed particles without additive; and ^(3)^ compared with the particles without co-processing.

**Table 6:** Pros and cons of modifiers.

Modifier	Pros	Cons
HPMC	(1) High glass transition temperature (170–180 °C) and viscosity; excellent bonding capability, tableting performance, and crystal growth inhibitor; (2) low hygroscopicity and surface tension for aqueous solutions; (3) non-toxic and improves stability.	(1) Long disintegration time.
PVP	(1) High glass transition temperature (160 °C); excellent solubility, plastic deformability, biocompatibility, and crystal growth inhibitor; (2) Almost no effect on the disintegration time of tablets; (3) non-toxic and increases stability.	(1) High hygroscopicity.
SiO_2_	(1) High porosity, surface area, surface free energy, and drug loading; excellent flowability, dispersion, and biocompatibility; safety; (2) Low cohesion, sticking and static effect; and (3) can be classified into hydrophilic and hydrophobic groups.	(1) Low density.
MCC	(1) Good plastic deformation, compressibility, and compactibility; excellent dilution capacity and disintegration behavior.	/.
Mannitol	(1) Amorphous state is conducive to improve disintegration, crystallizing excipient; (2) low viscosity; and (3) edible.	(1) Crystal state is bad for disintegration.
PVPP	(1) excellent plastic deformability, biocompatibility, and crystal growth inhibitor, (2) super disintegrant for orally disintegrating tablets.	(1) Poor flowability.
Ammonium bicarbonate	(1) Porous agent; and (2) improves the disintegration of tablets.	(1) Unstable chemical substance.

## Data Availability

Not applicable.
